# Quenching Kinetics of Triplet-State Photosensitizers in Environmental Photochemistry

**DOI:** 10.1021/acs.est.6c05761

**Published:** 2026-09-10

**Authors:** Sha Zhu, Keying Fan, Davide Vione, Yiwu Tang, Richard Spinney, Jannis Wenk, Ruiyang Xiao

**Affiliations:** † Institute of Environmental Engineering, School of Metallurgy and Environment, 12570 Central South University, Changsha 410083, China; ‡ Chinese National Engineering Research Center for Control & Treatment of Heavy Metal Pollution, Changsha 410083, China; § Department of Chemistry, University of Turin, Via Pietro Giuria 5, Torino 10125, Italy; ∥ Department of Chemistry and Biochemistry, 2647 The Ohio State University, Columbus, Ohio 43210, United States; ⊥ Department G1—General Water Quality Issues, Federal Institute of Hydrology, Division G—Qualitative Hydrology, Am Mainzer Tor 1, Koblenz 56068, Germany; # Institute for Integrated Natural Sciences, Faculty 3: Mathematics/Natural Sciences, 1555 University of Koblenz, Universitätsstraße 1, Koblenz 56070, Germany; ¶ Department of Chemical Engineering, University of Bath, Claverton Down, Bath, Bath BA2 7AY, U.K.

**Keywords:** quenching rate constant, dissolved organic matter, triplet excited state, laser flash photolysis, proton-coupled electron transfer, energy transfer, structure–reactivity relationship, environmental photochemistry

## Abstract

Triplet-state photosensitizers (^3^Sens*) are central to carbon cycling and pollutant degradation in sunlit surface and atmospheric waters, but their environmental roles remain only partly understood. Although the photophysical and photochemical properties of ^3^Sens* have been investigated, most studies remain confined to simplified systems with limited environmental relevance. This review critically analyzes approaches for characterizing the kinetic behavior of ^3^Sens*, focusing on bimolecular quenching rate constants (*k*_q_, M^–1^ s^–1^), and evaluates the strengths and limitations of current methodologies for their determination. A curated dataset (*n* = 105), spanning 5 orders of magnitude (10^5^ to 10^9^ M^–1^ s^–1^), is then used to analyze how molecular structure (electron-donating groups, conjugation, steric hindrance) and environmental conditions (pH, solvent polarity, ionic strength, temperature) govern *k*_q_ and alter underlying reaction mechanisms. By coupling kinetic isotope effects with spectroscopic evidence, the review distinguishes redox-driven pathways from energy transfer processes. These insights connect molecular-scale controls on *k*_q_ with environmentally relevant conditions. However, key gaps remain in triplet-yield data, mixed-photosensitizer systems, and interfacial processes limiting our understanding of ^3^Sens*-mediated transformations in aqueous and atmospheric multiphase environments.

## Graphical abstract

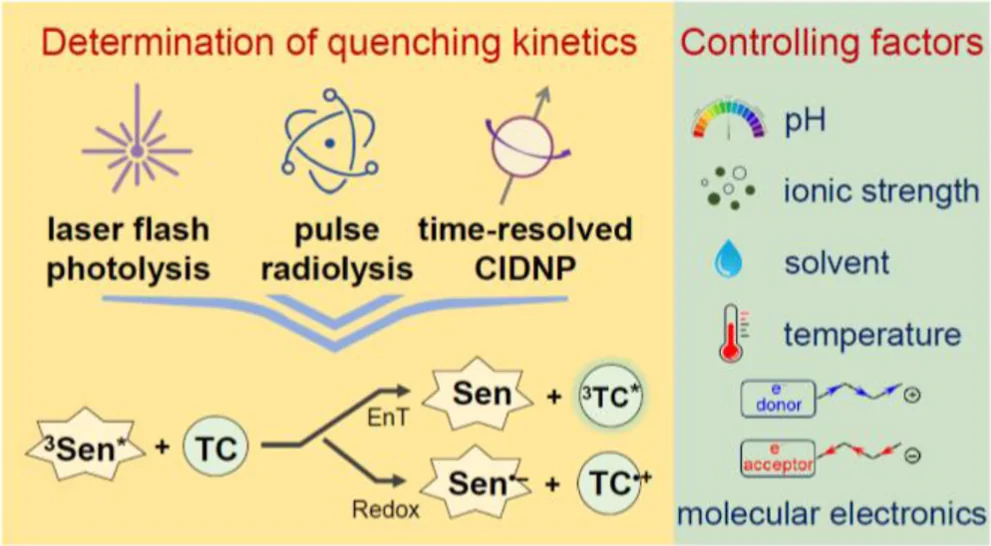

## 1 Introduction

Photochemical processes mediate the transformation of pollutants across sunlit environmental compartments, spanning from surface waters to the troposphere.^1,2^ These light-driven reactions proceed through various reactive intermediates that ultimately determine the fate of pollutants and their environmental impact. Among such intermediates, the excited triplet states of photosensitizers (^3^Sens*) are particularly important. With lifetimes extending into the microsecond range and substantial intrinsic reactivity, ^3^Sens* serve as major drivers in numerous transformation pathways.^3,4^ In sunlit surface waters, ^3^Sens* mediate the transformation of otherwise recalcitrant pollutants, including pesticides, antibiotics, and endocrine disruptors.^5,6^ Their impact extends beyond aquatic systems to atmospheric environments, where they contribute to the transformation of both volatile and particulate organic compounds.^7,8^ Atmospheric reactions initiated by ^3^Sens* contribute to secondary organic aerosol formation, promote halogen activation, and accelerate photochemical aging of organic aerosols, processes that ultimately influence air quality and global climate.^9,10^

When organic molecules absorb sunlight, a cascade of electronic transitions determines their subsequent reactive potential. Figure 1 briefly illustrates this process: Initial photon absorption elevates a Sens molecule to its lowest excited singlet-state (^1^Sens*), which is short-lived (10^–12^ to 10^–9^ s), and may subsequently undergo intersystem crossing (ISC) to produce the longer-lived excited triplet-state Sens (^3^Sens*), with lifetimes in the range of 10^–6^ to 10^–3^ s.^11,12^ Sens possessing conjugated π-systems, including aromatic hydrocarbons, dyes, and natural chromophores, readily form ^3^Sens* due to efficient ISC and favorable electronic configurations. Once populated, ^3^Sens* can either return to ground state through photophysical pathways via radiative and non-radiative transitions^13^ or participate in chemical reactions that transform pollutants. While short-lived ^1^Sens* rapidly deactivate with negligible environmental impact,^14^ the longer-lived ^3^Sens* allow sufficient time for pollutant interactions. These interactions proceed through both direct and indirect pathways: ^3^Sens* can directly oxidize or reduce pollutants,^4^ or generate reactive intermediates including radical species such as hydroxyl radical (^•^OH) and superoxide (O_2_^•–^),^15,16^ and non-radical species such as singlet oxygen (^1^O_2_) and hydrogen peroxide (H_2_O_2_)^17,18^ that further propagate transformation processes.

**Figure 1:**
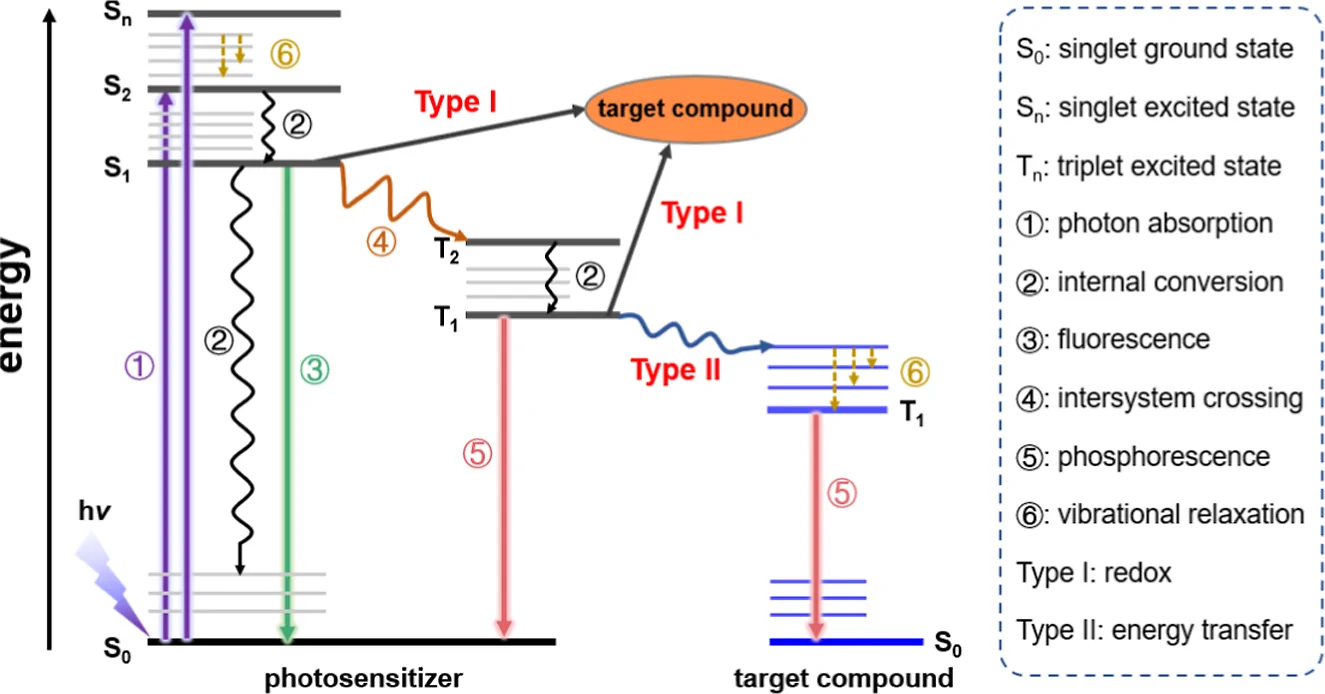
Photophysical and photochemical processes of a photosensitizer upon photon absorption illustrated by a simplified Jablonski diagram. The excited-state photosensitizer can interact with target compound through either electron transfer in the type I pathway or energy transfer in the type II pathway.

At the molecular level, photochemical reactions of ^3^Sens* with target compounds (TCs) proceed through two principal mechanisms: energy transfer (EnT) and redox reactions.^19^ EnT from ^3^Sens* to TCs may occur when the excitation energy of the ^3^Sens* matches or exceeds that of the TCs. In aquatic and atmospheric systems, the triplet excitation energy (*E*_T_) of Sens typically falls within 30–70 kcal mol^–1^.^14^ This energy parameter defines a reactivity spectrum among environmental Sens: polycyclic aromatic hydrocarbons (PAHs)-like moieties and quinones typically produce lower-energy triplets, whereas aromatic ketones and other carbonyl-containing compounds (*e.g.*, coumarins and chromones) form higher-energy triplets with correspondingly broader reactivity profiles. A broader compilation of *E*_T_ values and excited-state redox properties for DOM model Sens and other widely used Sens, together with the inferred distribution of ^3^DOM* energies, is available in the review by McNeill and Canonica.^14^ Representative model compounds illustrate this mechanistic distinction. 4-Carboxybenzophenone (CBBP, *E*_T_ = 68 kcal mol^–1^), a proxy for triplet dissolved organic matter (DOM), predominantly engages in EnT with TCs with lower *E*_T_ values.^14,20^ Conversely, methylene blue (^3^MB*, *E*_T_ = 33 kcal mol^–1^),^13^ functions predominantly as an electron acceptor and undergoes redox reactions with substituted phenols and anilines.^21^ Thus, *E*_T_ provides a useful first-pass thermodynamic criterion for pathway assignment. Triplet–triplet EnT is feasible when the *E*_T_ of ^3^Sens* is sufficiently high relative to that of the TC. When this condition is not satisfied, or when redox pathways are also thermodynamically accessible, photoinduced electron transfer (PET) can be evaluated from the reaction free energy calculated using redox potentials and the Rehm–Weller relationship. Proton-coupled electron transfer (PCET) and hydrogen atom transfer (HAT) require additional consideration of protonation state, p*K*_a_, hydrogen-bonding interactions, and X–H bond dissociation energies. Among the thermodynamically feasible pathways, the dominant mechanism should be assigned by combining measured *k*_q_ values with transient spectral evidence, kinetic isotope effects, and product analysis.

The bimolecular quenching rate constant (*k*_q_, M^–1^ s^–1^) serves as the primary metric for evaluating how rapidly ^3^Sens* interact with TCs. Experimentally, *k*_q_ can be determined by fitting observed decay rates to the Stern–Volmer relationship^22,23^

1 $$k_{obs}=k_{0}+k_{q}\left[TC\right]$$

where *k*_obs_ represents the pseudo-first-order decay rate constant of ^3^Sens* (s^–1^), and *k*_0_ is the first-order decay rate constant (s^–1^) in the absence of TCs. Reported *k*_q_ values for ^3^Sens* reactions span several orders of magnitude (10^5^ to 10^9^ M^–1^ s^–1^).^13,24^ Such a broad range reflects that ^3^Sens* quenching is governed by the combined influence of multiple molecular characteristics and environmental conditions, including pH, solvent properties, ionic strength, and temperature.^25^ Even structurally similar aromatic compounds may exhibit large differences in *k*_q_ because subtle electronic differences alter their capacity to donate or accept electrons.^21^ In parallel, environmental conditions can change encounter dynamics and reaction free energies, creating additional variability in observed *k*_q_ values.^26^ Understanding these interconnected factors is essential for interpreting ^3^Sens* kinetics and understanding the downstream photochemical pathways that determine pollutant transformation.

Early contributions to environmental photochemistry established ^3^Sens* as fundamental drivers in the oxidation of aquatic organic pollutants.^27–29^ Building on this foundation, McNeill and Canonica provided a mechanistic and photophysical framework for ^3^DOM*,^14^ establishing the core concepts that have guided subsequent investigations. Later, a dedicated review examined the formation pathways of organic ^3^Sens* and their involvement in various environmental processes.^30^ More recently, attention has shifted toward analytical development, particularly probe-based detection methods for ^3^DOM*.^6^ Despite these advances, no existing review has systematically assessed the quenching kinetics of ^3^Sens*, especially the *k*_q_ values that are essential for quantifying pollutant transformation. The present review addresses this gap by examining the major experimental techniques for determining *k*_q_ values and by identifying the molecular and environmental factors (*e*.*g*., pH, solvent properties, ionic strength, temperature) that shape these reactions. In addition, a curated dataset of 105 *k*_q_ values for ^3^Sens* reacting with structurally diverse organic TCs in aqueous systems was critically compiled, providing a quantitative foundation for comparison. By integrating kinetic measurements with mechanistic interpretation, this review establishes the basis for linking light-driven quenching dynamics to pollutant fate in both natural and engineered environments.

## 2 Experimental Platforms for Quenching Kinetics

Accurate determination of excited-state lifetimes is fundamental for evaluating quenching kinetics. In bimolecular reactions between ^3^Sens* and TCs, the observed lifetime of ^3^Sens* decreases with increasing TC concentration, providing a direct means of extracting *k*_q_ from time-resolved decay data. The lifetimes of short-lived excited states are typically determined using time-resolved techniques, including laser flash photolysis (LFP), pulse radiolysis (PR), and time-resolved chemically induced dynamic nuclear polarization (TR-CIDNP). Despite differences in instrumentation, they share a common principle: An excitation source initiates the excited state, followed by time-resolved detection of its decay. Specifically, LFP utilizes a laser pulse as the excitation source and monitors changes in transient absorbance using a monochromatic probe beam. PR employs high-energy electron pulses, while TR-CIDNP involves laser-initiated excitation and detection via nuclear magnetic resonance (NMR). The three methods discussed below have been applied primarily to bulk aqueous solutions and atmospheric aqueous phases, including cloud droplets, fog water, and aerosol liquid water. In this study, atmospheric systems therefore refer mainly to condensed aqueous microenvironments rather than true gas-phase chemistry. Table 1 summarizes the merits and limitations of each method, while schematic diagrams (Figure 2) are provided to aid conceptual understanding.

**Table 1:** Comparison of Laser Flash Photolysis, Pulse Radiolysis, and Time-Resolved Chemically Induced Dynamic Nuclear Polarization for Quenching Rate Constant (*k*_q_) Determination

technique	advantages	disadvantages
LFP	high temporal resolution	requires Sens with sufficient absorption at specific wavelength
	monochromatic source	
PR	generates high concentrations of reactive intermediates	non-uniform energy deposition within the sample
	applicable to a wide range of sample types and matrices	requires access to a specialized radiation facility
		solvent radiolysis can interfere with product analysis
TR-CIDNP	resolves spectral overlap between Sens and radical products	restricted to reactions proceeding via radical pairs
	directly tracks radical-pair dynamics	accuracy depends on the quality and calibration of the reference compound *k*_q_

**Figure 2:**
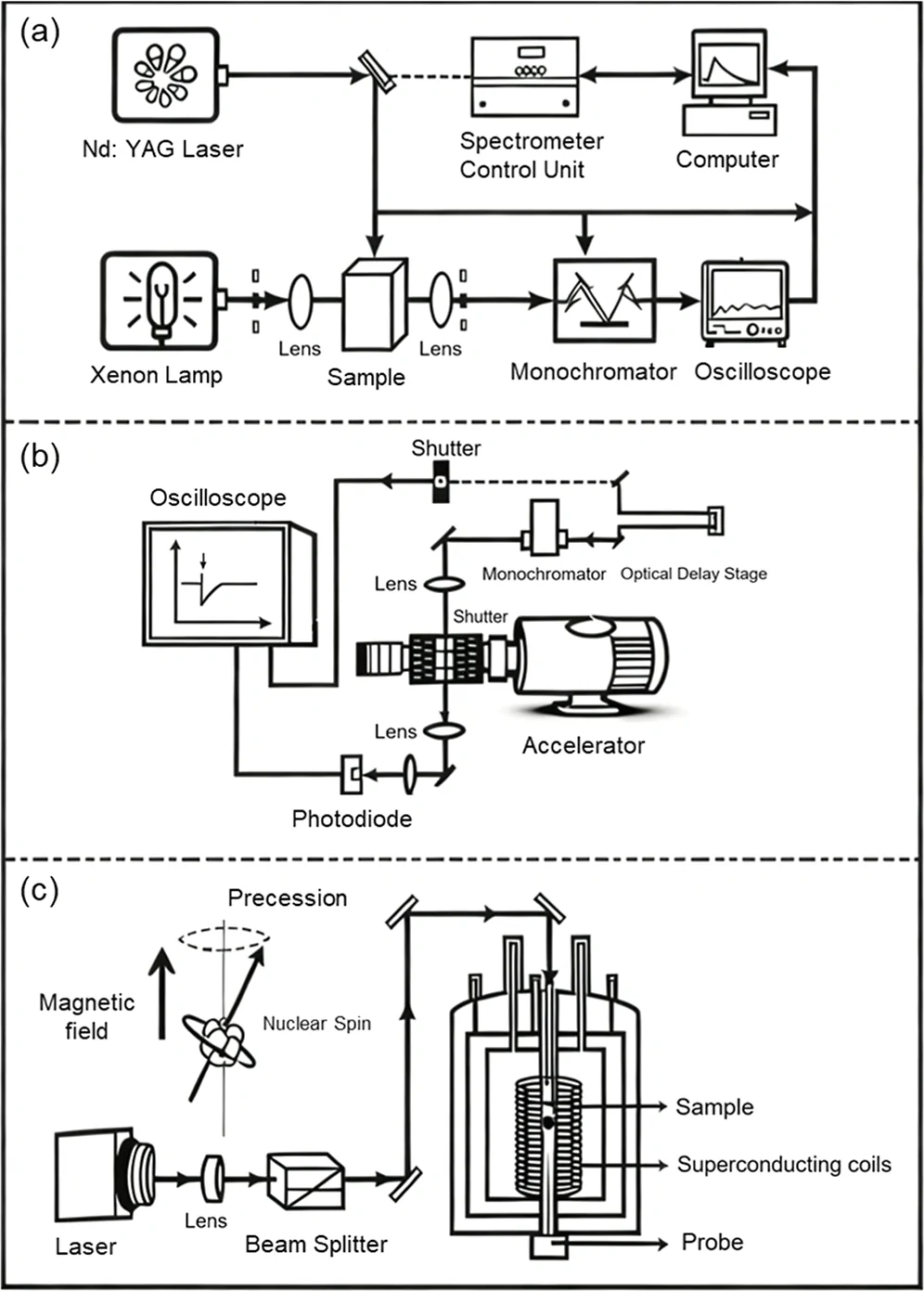
Principles of laser flash photolysis (a), pulse radiolysis (b) and time-resolved chemically induced dynamic nuclear polarization (c).

### 2.1 Laser Flash Photolysis

LFP is a widely used time-resolved technique for studying the kinetics of ^3^Sens*.^31^ It represents a laser-based implementation of flash photolysis, within the broader family of transient absorption spectroscopy (TAS),^32^ which encompasses both ultrafast pump–probe spectroscopy (femtosecond to picosecond resolution) and flash photolysis (nanosecond resolution).^33,34^

LFP operates by delivering short, high-energy pulses (typically 10–100 mJ per pulse), to excite ground-state Sens, then using a continuous monochromatic light source (single wavelength with narrow bandwidth) to precisely detect transient species absorbance changes. With ^3^Sens* lifetimes typically ranging from nanoseconds to microseconds, the high temporal resolution (down to ∼10^–8^ to 10^–9^ s) of LFP is particularly suitable for these measurements.^35^

Figure 3 illustrates the LFP pump–probe setup and the resulting change in absorbance (Δ*A*), measured as the difference in transmitted probe intensity before and after excitation. This change in absorbance serves as the basis for kinetic analysis, allowing real-time monitoring of transient species formation and decay.^36^ When a TC interacts with a ^3^Sens*, dynamic quenching introduces a non-radiative decay pathway that accelerates deactivation. By measuring Δ*A* decay at various TC concentrations, *k*_obs_ in eq 1 can be determined through exponential fitting. Plotting *k*_obs_ against [TC] then allows for the determination of the *k*_q_ value.

**Figure 3:**
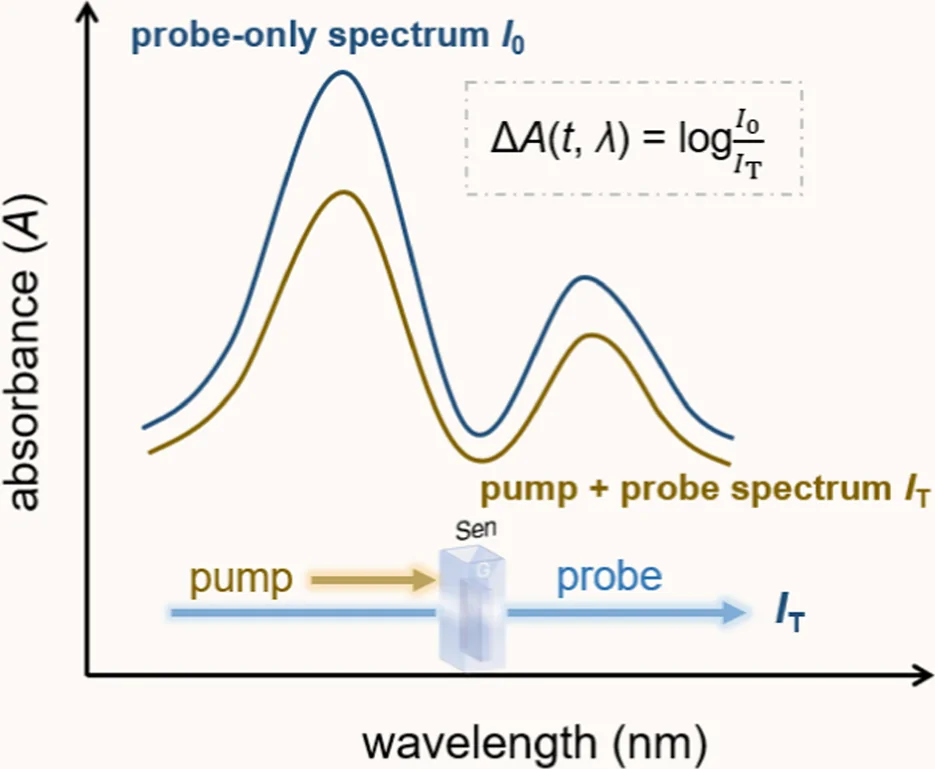
Schematic representation of the pump–probe setup and the corresponding change in absorbance (Δ*A*). *I*_0_ is the “probe-only” absorption intensity that is measured before the pump pulse, and *I*_T_ is the time dependent “pump + probe” intensity at a specific wavelength λ.

LFP is highly versatile for investigating excited-state quenching kinetics in different environmental systems. Table 2 demonstrates its dominance in quantifying *k*_q_ values for interactions between ^3^Sens* and various TCs, with LFP used for 97 of the 105 entries. Investigated compounds include electron-rich aromatics (substituted phenols, anilines, and quinones) commonly found in natural and engineered aquatic environments. The nanosecond temporal resolution of LFP makes it well-suited to study diffusion-controlled quenching under environmentally relevant conditions.

**Table 2:** Quenching Rate Constants (*k*_q_, M^–1^ s^–1^) of Triplet-State Photosensitizers (^3^Sens*) with Organic Target Compounds (TCs) in Aqueous Solution^a^

#	Sens	TCs	*k*_q_ (M^–1^ s^–1^)	refs	comment
1	2-acetonaphthone (2-AN)	2,4,6-trimethylphenol	(7.2 ± 0.1) × 10^8^	^120^	LFP, DK, excited at 308 or 351 nm, monitored at 440 nm, *k*_0_ = 1.67 × 10^4^ s^–1^, aerated aqueous solution containing 2-AN (0.05∼0.2 mM) and TC (0.5∼2 mM), phosphate buffer (50 mM), pH = 8.0, at RT
2	2-acetonaphthone (2-AN)	3,4-dimethoxyphenol	(3.1 ± 0.1) × 10^9^	^120^	LFP, DK, excited at 308 or 351 nm, monitored at 440 nm, k0 = 1.67 × 10^4^ s^–1^, aerated aqueous solution containing 2-AN (0.05∼0.2 mM) and TC (0.5∼2 mM), phosphate buffer (50 mM), pH = 8.0, at RT
3	2-acetonaphthone (2-AN)	4-cyanophenol	(1.3 ± 1.3) × 10^7^	^120^	LFP, DK, excited at 308 or 351 nm, monitored at 440 nm, *k*_0_ = 1.67 × 10^4^ s^–1^, aerated aqueous solution containing 2-AN (0.05∼0.2 mM) and TC (0.5∼2 mM), phosphate buffer (50 mM), pH = 6.0, at RT
4	2-acetonaphthone (2-AN)	4-hydroxy-benzoic acid	(2.6 ± 1.3) × 10^7^	^120^	LFP, DK, excited at 308 or 351 nm, monitored at 440 nm, *k*_0_ = 1.67 × 10^4^ s^–1^, aerated aqueous solution containing 2-AN (0.05∼0.2 mM) and TC (0.5∼2 mM), phosphate buffer (50 mM), pH = 7.0, at RT
5	2-acetonaphthone (2-AN)	4-methoxyphenol	(1.8 ± 0.1) × 10^9^	^120^	LFP, DK, excited at 308 or 351 nm, monitored at 440 nm, *k*_0_ = 1.67 × 10^4^ s^–1^, aerated aqueous solution containing 2-AN (0.05∼0.2 mM) and TC (0.5∼2 mM), phosphate buffer (50 mM), pH = 8.0, at RT
6	2-acetonaphthone (2-AN)	4-methylphenol	(8.4 ± 0.3) × 10^7^	^120^	LFP, DK, excited at 308 or 351 nm, monitored at 440 nm, *k*_0_ = 1.67 × 10^4^ s^–1^, aerated aqueous solution containing 2-AN (0.05∼0.2 mM) and TC (0.5∼2 mM), phosphate buffer (50 mM), pH = 8.0, at RT
7	2-acetonaphthone (2-AN)	phenol	(3.3 ± 1.3) × 10^7^	^120^	LFP, DK, excited at 308 or 351 nm, monitored at 440 nm, *k*_0_ = 1.67 × 10^4^ s^–1^, aerated aqueous solution containing 2-AN (0.05∼0.2 mM) and TC (0.5∼2 mM), phosphate buffer (50 mM), pH = 8.0, at RT
8	2-acetonaphthone (2-AN)	trolox	(2.7 ± 0.2) × 10^9^	^120^	LFP, DK, excited at 308 or 351 nm, monitored at 440 nm, *k*_0_ = 1.67 × 10^4^ s^–1^, aerated aqueous solution containing 2-AN (0.05∼0.2 mM) and TC (0.5∼2 mM), phosphate buffer (50 mM), pH = 8.0, at RT
9	2-acetonaphthone (2-AN)	tyrosine	(3.7 ± 1.3) × 10^7^	^120^	LFP, DK, excited at 308 or 351 nm, monitored at 440 nm, *k*_0_ = 1.67 × 10^4^ s^–1^, aerated aqueous solution containing 2-AN (0.05∼0.2 mM) and TC (0.5∼2 mM), phosphate buffer (50 mM), pH = 7.0, at RT
10	3,3′,4,4′-benzophenone tetracarboxylic acid (BPTC)	adenine	2.3 × 10^9^	^65^	LFP, DK, excited at 308 nm, monitored at 550 nm, Ar-saturated aqueous solution containing BPTC (0.1 mM) and TC (0∼1.5 mM), phosphate buffer (10 mM), 4.7 < pH < 9.9, at RT
11	3,3′,4,4′-benzophenone tetracarboxylic acid (BPTC)	adenosine	4.0 × 10^9^	^65^	LFP, DK, excited at 308 nm, monitored at 590 nm, Ar-saturated aqueous solution containing BPTC (0.1 mM) and TC (0∼1.5 mM), phosphate buffer (10 mM), 3.5 < pH < 4.7, at RT
12	3,3′,4,4′-benzophenone tetracarboxylic acid (BPTC)	guanosine-5′-monophosphate	2.6 × 10^8^	^66^	LFP, DK, excited at 308 nm, monitored at 550 nm, *k*_0_ = 1.25 × 10^5^ s^–1^ est., Ar-saturated aqueous solution containing BPTC (0.1 mM) and TC (0∼0.67 mM), phosphate buffer (10 mM), pH = 6.8, at RT
13	3,3′,4,4′-benzophenone tetracarboxylic acid (BPTC)	thymidine	4.0 × 10^8^	^65^	LFP, DK, excited at 308 nm, monitored at 590 nm, *k*_0_ = 6.67 × 10^4^ s^–1^ est., Ar-saturated aqueous solution containing BPTC (0.1 mM) and TC (0∼1.5 mM), phosphate buffer (10 mM), pH = 6.4, at RT
14	3,3′,4,4′-benzophenone tetracarboxylic acid (BPTC)	thymine	1.0 × 10^9^	^65^	LFP, DK, excited at 308 nm, monitored at 550 nm, Ar-saturated aqueous solution containing BPTC (0.1 mM) and TC (0∼1.5 mM), phosphate buffer (10 mM), 4.7 < pH < 9.9, at RT
15	3-methoxyacetophenone (3-MAP)	2,4,6-trimethylphenol	(2.6 ± 0.3) × 10^9^	^120^	LFP, DK, excited at 308 or 351 nm, monitored at 395 nm, *k*_0_ = 2.0 × 10^4^ s^–1^, aerated aqueous solution containing 3-MAP (0.05∼0.2 mM) and TC (0.5∼2 mM), phosphate buffer (50 mM), pH = 8.0, at RT
16	3-methoxyacetophenone (3-MAP)	3,4-dimethoxyphenol	(2.4 ± 0.3) × 10^9^	^120^	LFP, DK, excited at 308 or 351 nm, monitored at 395 nm, *k*_0_ = 2.0 × 10^4^ s^–1^, aerated aqueous solution containing 3-MAP (0.05∼0.2 mM) and TC (0.5∼2 mM), phosphate buffer (50 mM), pH = 8.0, at RT
17	3-methoxyacetophenone (3-MAP)	4-cyanophenol	(1.2 ± 0.5) × 10^8^	^120^	LFP, DK, excited at 308 or 351 nm, monitored at 395 nm, *k*_0_ = 2.0 × 10^4^ s^–1^, aerated aqueous solution containing 3-MAP (0.05∼0.2 mM) and TC (0.5∼2 mM), phosphate buffer (50 mM), pH = 6.0, at RT
18	3-methoxyacetophenone (3-MAP)	4-hydroxy-benzoic acid	(4.6 ± 0.6) × 10^8^	^120^	LFP, DK, excited at 308 or 351 nm, monitored at 395 nm, *k*_0_ = 2.0 × 10^4^ s^–1^, aerated aqueous solution containing 3-MAP (0.05∼0.2 mM) and TC (0.5∼2 mM), phosphate buffer (50 mM), pH = 7.0, at RT
19	3-methoxyacetophenone (3-MAP)	4-methoxyphenol	(2.7 ± 0.3) × 10^9^	^120^	LFP, DK, excited at 308 or 351 nm, monitored at 395 nm, *k*_0_ = 2.0 × 10^4^ s^–1^, aerated aqueous solution containing 3-MAP (0.05∼0.2 mM) and TC (0.5∼2 mM), phosphate buffer (50 mM), pH = 8.0, at RT
20	3-methoxyacetophenone (3-MAP)	4-methylphenol	(3.0 ± 0.2) × 10^9^	^120^	LFP, DK, excited at 308 or 351 nm, monitored at 395 nm, *k*_0_ = 2.0 × 10^4^ s^–1^, aerated aqueous solution containing 3-MAP (0.05∼0.2 mM) and TC (0.5∼2 mM), phosphate buffer (50 mM), pH = 8.0, at RT
21	3-methoxyacetophenone (3-MAP)	phenol	(5.1 ± 0.4) × 10^8^	^120^	LFP, DK, excited at 308 or 351 nm, monitored at 395 nm, *k*_0_ = 2.0 × 10^4^ s^–1^, aerated aqueous solution containing 3-MAP (0.05∼0.2 mM) and TC (0.5∼2 mM), phosphate buffer (50 mM), pH = 8.0, at RT
22	3-methoxyacetophenone (3-MAP)	trolox	(2.2 ± 0.2) × 10^9^	^120^	LFP, DK, excited at 308 or 351 nm, monitored at 395 nm, *k*_0_ = 2.0 × 10^4^ s^–1^, aerated aqueous solution containing 3-MAP (0.05∼0.2 mM) and TC (0.5∼2 mM), phosphate buffer (50 mM), pH = 8.0, at RT
23	3-methoxyacetophenone (3-MAP)	tyrosine	(6.6 ± 0.8) × 10^8^	^120^	LFP, DK, excited at 308 or 351 nm, monitored at 395 nm, *k*_0_ = 2.0 × 10^4^ s^–1^, aerated aqueous solution containing 3-MAP (0.05∼0.2 mM) and TC (0.5∼2 mM), phosphate buffer (50 mM), pH = 7.0, at RT
24	4-thiouridine	cysteine	4.0 × 10^8^	^121^	LFP, DK, excited at 353 nm, monitored at 530 nm, *k*_0_ = 5.6 × 10^6^ s^–1^, Sens concentration: NR, TC concentration: NR, at RT
25	4-thiouridine	histidine	4.0 × 10^7^	^121^	LFP, DK, excited at 353 nm, monitored at 530 nm, *k*_0_ = 5.6 × 10^6^ s^–1^, Sens concentration: NR, TC concentration: NR, at RT
26	4-thiouridine	lysine	4.5 × 10^8^	^121^	LFP, DK, excited at 353 nm, monitored at 530 nm, *k*_0_ = 5.6 × 10^6^ s^–1^, Sens concentration: NR, TC concentration: NR, at RT
27	4-thiouridine	methionine	1.5 × 10^9^	^121^	LFP, DK, excited at 353 nm, monitored at 530 nm, *k*_0_ = 5.6 × 10^6^ s^–1^, Sens concentration: NR, TC concentration: NR, at RT
28	4-thiouridine	orotic acid	1.5 × 10^9^	^121^	LFP, DK, excited at 353 nm, monitored at 530 nm, *k*_0_ = 5.6 × 10^6^ s^–1^, Sens concentration: NR, TC concentration: NR, at RT
29	4-thiouridine	thymidine	7.0 × 10^8^	^121^	LFP, DK, excited at 353 nm, monitored at 530 nm, *k*_0_ = 5.6 × 10^6^ s^–1^, Sens concentration: NR, TC concentration: NR, at RT
30	4-thiouridine	tryptophan	1.5 × 10^9^	^121^	LFP, DK, excited at 353 nm, monitored at 530 nm, *k*_0_ = 5.6 × 10^6^ s^–1^, Sens concentration: NR, TC concentration: NR, at RT
31	4-thiouridine	tyrosine	2.0 × 10^9^	^121^	LFP, DK, excited at 353 nm, monitored at 530 nm, *k*_0_ = 5.6 × 10^6^ s^–1^, Sens concentration: NR, TC concentration: NR, at RT
32	4-thiouridine	uracil	2.0 × 10^8^	^121^	LFP, DK, excited at 353 nm, monitored at 530 nm, *k*_0_ = 5.6 × 10^6^ s^–1^, Sens concentration: NR, TC concentration: NR, at RT
33	4-thiouridine	uridine	2.7 × 10^8^	^121^	LFP, DK, excited at 353 nm, monitored at 530 nm, *k*_0_ = 5.6 × 10^6^ s^–1^, Sens concentration: NR, TC concentration: NR, at RT
34	Acetone	*N*,*N*-dimethylaniline	(2.5 ± 0.8) × 10^9^	^122^	LFP, DK, excited at 265 nm, degassed aqueous solution containing acetone (50 mM) and TC, TC concentration: NR, *T* = 296 K
35	acetone	*n*-hexylamine	(1.0 ± 0.6) × 10^7^	^122^	LFP, DK, excited at 265 nm, degassed aqueous solution containing acetone (50 mM) and TC, TC concentration: NR, *T* = 296 K
36	acetone	*t*-butylamine	(1.9 ± 0.4) × 10^6^	^122^	LFP, DK, excited at 265 nm, degassed aqueous solution containing acetone (50 mM) and TC, TC concentration: NR, *T* = 296 K
37	acetone	triethylamine	(2.7 ± 0.5) × 10^8^	^122^	LFP, DK, excited at 265 nm, degassed aqueous solution containing acetone (50 mM) and TC, TC concentration: NR, *T* = 296 K
38	benzophenone (BP)	2,4,6-trimethylphenol	(5.1 ± 0.9) × 10^9^	^120^	LFP, DK, excited at 308 or 351 nm, monitored at 520 nm, *k*_0_ = 5.0 × 10^4^ s^–1^, aerated aqueous solution containing BP (0.05∼0.2 mM) and TC (0.5∼2 mM), phosphate buffer (50 mM), pH = 8.0, at RT
39	benzophenone (BP)	2-mercaptoethanol	1.7 × 10^9^	^24^	LFP, DK, excited at 337 nm, monitored at 540 nm, *k*_0_ = 3.33 × 10^4^ s^–1^, N_2_-saturated aqueous solution containing BP (0.01 mM) and TC (∼0.1 mM), phosphate buffer (50 mM), pH = 7.0, at RT
40	benzophenone (BP)	3,4-dimethoxyphenol	(5.2 ± 0.2) × 10^9^	^120^	LFP, DK, excited at 308 or 351 nm, monitored at 520 nm, *k*_0_ = 5.0 × 10^4^ s^–1^, aerated aqueous solution containing BP (0.05∼0.2 mM) and TC (0.5∼2 mM), phosphate buffer (50 mM), pH = 8.0, at RT
41	benzophenone (BP)	4-cyanophenol	(3.0 ± 0.3) × 10^9^	^120^	LFP, DK, excited at 308 or 351 nm, monitored at 520 nm, *k*_0_ = 5.0 × 10^4^ s^–1^, aerated aqueous solution containing BP (0.05∼0.2 mM) and TC (0.5∼2 mM), phosphate buffer (50 mM), pH = 6.0, at RT
42	benzophenone (BP)	4-hydroxy-benzoic acid	(2.9 ± 0.3) × 10^9^	^120^	LFP, DK, excited at 308 or 351 nm, monitored at 520 nm, *k*_0_ = 5.0 × 10^4^ s^–1^, aerated aqueous solution containing BP (0.05∼0.2 mM) and TC (0.5∼2 mM), phosphate buffer (50 mM), pH = 7.0, at RT
43	benzophenone (BP)	4-methoxyphenol	(4.2 ± 0.6) × 10^9^	^120^	LFP, DK, excited at 308 or 351 nm, monitored at 520 nm, *k*_0_ = 5.0 × 10^4^ s^–1^, aerated aqueous solution containing BP (0.05∼0.2 mM) and TC (0.5∼2 mM), phosphate buffer (50 mM), pH = 8.0, at RT
44	benzophenone (BP)	4-methylphenol	(4.2 ± 0.2) × 10^9^	^120^	LFP, DK, excited at 308 or 351 nm, monitored at 520 nm, *k*_0_ = 5.0 × 10^4^ s^–1^, aerated aqueous solution containing BP (0.05∼0.2 mM) and TC (0.5∼2 mM), phosphate buffer (50 mM), pH = 8.0, at RT
45	benzophenone (BP)	cysteine	3.2 × 10^8^	^24^	LFP, DK, excited at 337 nm, monitored at 540 nm, *k*_0_ = 3.33 × 10^4^ s^–1^, N_2_-saturated aqueous solution containing BP (0.01 mM) and TC (∼0.1 mM), phosphate buffer (50 mM), pH = 7.0, at RT
46	benzophenone (BP)	cystine	5.2 × 10^8^	^24^	LFP, DK, excited at 337 nm, monitored at 540 nm, *k*_0_ = 3.33 × 10^4^ s^–1^, N_2_-saturated aqueous solution containing BP (0.01 mM) and TC (∼0.1 mM), phosphate buffer (50 mM), pH = 7.0, at RT
47	benzophenone (BP)	dl-penicillamine	5.8 × 10^8^	^24^	LFP, DK, excited at 337 nm, monitored at 540 nm, *k*_0_ = 3.33 × 10^4^ s^–1^, N_2_-saturated aqueous solution containing BP (0.01 mM) and TC (∼0.1 mM), phosphate buffer (50 mM), pH = 7.0, at RT
48	benzophenone (BP)	glutamine	3.0 × 10^6^	^24^	LFP, DK, excited at 337 nm, monitored at 540 nm, *k*_0_ = 3.33 × 10^4^ s^–1^, N_2_-saturated aqueous solution containing BP (0.01 mM) and TC (∼0.1 mM), phosphate buffer (50 mM), pH = 7.0, at RT
49	benzophenone (BP)	glutathione	6.7 × 10^8^	^24^	LFP, DK, excited at 337 nm, monitored at 540 nm, *k*_0_ = 3.33 × 10^4^ s^–1^, N_2_-saturated aqueous solution containing BP (0.01 mM) and TC (∼0.1 mM), phosphate buffer (50 mM), pH = 7.0, at RT
50	benzophenone (BP)	glycine	2.0 × 10^5^	^24^	LFP, DK, excited at 337 nm, monitored at 540 nm, *k*_0_ = 3.33 × 10^4^ s^–1^, N_2_-saturated aqueous solution containing BP (0.01 mM) and TC (∼0.1 mM), phosphate buffer (50 mM), pH = 7.0, at RT
51	benzophenone (BP)	metoxuron	(2.6 ± 0.2) × 10^9^	^123^	LFP, DK, monitored at 350 nm, *k*_0_ = 1.05 × 10^5^ s^–1^, aerated aqueous solution containing BP (0.0135 mM) and TC (0.5 mM), phosphate buffer (5 mM), pH = 8.0, at RT
52	benzophenone (BP)	oxidized glutathione	1.3 × 10^9^	^24^	LFP, DK, excited at 337 nm, monitored at 540 nm, *k*_0_ = 3.33 × 10^4^ s^–1^, N_2_-saturated aqueous solution containing BP (0.01 mM) and TC (∼0.1 mM), phosphate buffer (50 mM), pH = 7.0, at RT
53	benzophenone (BP)	phenol	(3.9 ± 0.7) × 10^9^	^120^	LFP, DK, excited at 308 or 351 nm, monitored at 520 nm, *k*_0_ = 5.0 × 10^4^ s^–1^, aerated aqueous solution containing BP (0.05∼0.2 mM) and TC (0.5∼2 mM), phosphate buffer (50 mM), pH = 8.0, at RT
54	benzophenone (BP)	trolox	(4.1 ± 0.2) × 10^9^	^120^	LFP, DK, excited at 308 or 351 nm, monitored at 520 nm, *k*_0_ = 5.0 × 10^4^ s^–1^, aerated aqueous solution containing BP (0.05∼0.2 mM) and TC (0.5∼2 mM), phosphate buffer (50 mM), pH = 8.0, at RT
55	benzophenone (BP)	tyrosine	(2.6 ± 0.2) × 10^9^	^120^	LFP, DK, excited at 308 or 351 nm, monitored at 520 nm, *k*_0_ = 5.0 × 10^4^ s^–1^, aerated aqueous solution containing BP (0.05∼0.2 mM) and TC (0.5∼2 mM), phosphate buffer (50 mM), pH = 7.0, at RT
56	difloxacin (DFX)	2′-deoxyguanosine-5′-monophosphate	6.92 × 10^7^	^124^	LFP, DK, excited at 355 nm, monitored at 620 nm, N_2_-saturated aqueous solution containing DFX (0.1 mM) and TC (10 mM), phosphate buffer (1 mM), pH = 7.0
57	difloxacin (DFX)	tyrosine	3.14 × 10^8^	^124^	LFP, DK, excited at 355 nm, monitored at 620 nm, N_2_-saturated aqueous solution containing DFX (0.1 mM) and TC (5 mM), phosphate buffer (1 mM), pH = 7.0
58	flavin mononucleotide (FMN)	glycyl-l-tyrosine	(1.0 ± 0.1) × 10^9^	^125^	LFP, DK, monitored at 680 nm, *k*_0_ = 1.30 × 10^5^ s^–1^, N_2_-saturated aqueous solution containing FMN (0.2 mM) and TC, TC concentration: NR, phosphate buffer, pH = 7.0, at RT
59	flavin mononucleotide (FMN)	l-cysteine	(2.8 ± 0.3) × 10^7^	^125^	LFP, DK, monitored at 680 nm, *k*_0_ = 1.30 × 10^5^ s^–1^, N_2_-saturated aqueous solution containing FMN (0.2 mM) and TC, TC concentration: NR, phosphate buffer, pH = 7.0, at RT
60	flavin mononucleotide (FMN)	L-ethionine	(2.6 ± 0.3) × 10^7^	^125^	LFP, DK, monitored at 680 nm, *k*_0_ = 1.30 × 10^5^ s^–1^, N_2_-saturated aqueous solution containing FMN (0.2 mM) and TC, TC concentration: NR, phosphate buffer, pH = 7.0, at RT
61	flavin mononucleotide (FMN)	l-histidine	(4.5 ± 0.5) × 10^7^	^125^	LFP, DK, monitored at 680 nm, *k*_0_ = 1.30 × 10^5^ s^–1^, N_2_-saturated aqueous solution containing FMN (0.2 mM) and TC, TC concentration: NR, phosphate buffer, pH = 7.0, at RT
62	flavin mononucleotide (FMN)	l-methionine	(1.7 ± 0.2) × 10^7^	^125^	LFP, DK, monitored at 680 nm, *k*_0_ = 1.30 × 10^5^ s^–1^, N_2_-saturated aqueous solution containing FMN (0.2 mM) and TC, TC concentration: NR, phosphate buffer, pH = 7.0, at RT
63	flavin mononucleotide (FMN)	l-tryptophan	(1.7 ± 0.2) × 10^9^	^125^	LFP, DK, monitored at 680 nm, *k*_0_ = 1.30 × 10^5^ s^–1^, N_2_-saturated aqueous solution containing FMN (0.2 mM) and TC, TC concentration: NR, phosphate buffer, pH = 7.0, at RT
64	methylene blue (MB)	3-chloroaniline	(0.11 ± 0.01) × 10^8^	^21^	LFP, DK, excited at 665 nm, monitored at 832 nm, *k*_0_ = 2.4 × 10^4^ s^–1^, N_2_-saturated aqueous solution containing MB^+^ (0.01 mM) and TC (0.1∼1.6 mM), borate buffer (10 mM), pH = 8.4, at RT
65	methylene blue (MB)	3-ethoxyaniline	(1.30 ± 0.03) × 10^9^	^21^	LFP, DK, excited at 665 nm, monitored at 832 nm, *k*_0_ = 2.4 × 10^4^ s^–1^, N_2_-saturated aqueous solution containing MB^+^ (0.01 mM) and TC (0.1∼1.6 mM), borate buffer (10 mM), pH = 8.4, at RT
66	methylene blue (MB)	3-ethylaniline	(1.54 ± 0.04) × 10^9^	^21^	LFP, DK, excited at 665 nm, monitored at 832 nm, *k*_0_ = 2.4 × 10^4^ s^–1^, N_2_-saturated aqueous solution containing MB^+^ (0.01 mM) and TC (0.1∼1.6 mM), borate buffer (10 mM), pH = 8.4, at RT
67	methylene blue (MB)	3-fluoroaniline	(0.11 ± 0.01) × 10^8^	^21^	LFP, DK, excited at 665 nm, monitored at 832 nm, *k*_0_ = 2.4 × 10^4^ s^–1^, N_2_-saturated aqueous solution containing MB^+^ (0.01 mM) and TC (0.1∼1.6 mM), borate buffer (10 mM), pH = 8.4, at RT
68	methylene blue (MB)	3-hydroxyaniline	(1.83 ± 0.01) × 10^9^	^21^	LFP, DK, excited at 665 nm, monitored at 832 nm, *k*_0_ = 2.4 × 10^4^ s^–1^, N_2_-saturated aqueous solution containing MB^+^ (0.01 mM) and TC (0.1∼1.6 mM), borate buffer (10 mM), pH = 8.4, at RT
69	methylene blue (MB)	3-methoxyaniline	(1.17 ± 0.04) × 10^9^	^21^	LFP, DK, excited at 665 nm, monitored at 832 nm, *k*_0_ = 2.4 × 10^4^ s^–1^, N_2_-saturated aqueous solution containing MB^+^ (0.01 mM) and TC (0.1∼1.6 mM), borate buffer (10 mM), pH = 8.4, at RT
70	methylene blue (MB)	3-methylaniline	(1.52 ± 0.07) × 10^9^	^21^	LFP, DK, excited at 665 nm, monitored at 832 nm, *k*_0_ = 2.4 × 10^4^ s^–1^, N_2_-saturated aqueous solution containing MB^+^ (0.01 mM) and TC (0.1∼1.6 mM), borate buffer (10 mM), pH = 8.4, at RT
71	methylene blue (MB)	4-aminoaniline	(4.24 ± 0.13) × 10^9^	^21^	LFP, DK, excited at 665 nm, monitored at 832 nm, *k*_0_ = 2.4 × 10^4^ s^–1^, N_2_-saturated aqueous solution containing MB^+^ (0.01 mM) and TC (0.1∼1.6 mM), borate buffer (10 mM), pH = 8.4, at RT
72	methylene blue (MB)	4-chloroaniline	(3.79 ± 0.16) × 10^8^	^21^	LFP, DK, excited at 665 nm, monitored at 832 nm, *k*_0_ = 2.4 × 10^4^ s^–1^, N_2_-saturated aqueous solution containing MB^+^ (0.01 mM) and TC (0.1 ∼ 1.6 mM), borate buffer (10 mM), pH = 8.4, at RT
73	methylene blue (MB)	4-fluoroaniline	(8.65 ± 0.16) × 10^8^	^21^	LFP, DK, excited at 665 nm, monitored at 832 nm, *k*_0_ = 2.4 × 10^4^ s^–1^, N_2_-saturated aqueous solution containing MB^+^ (0.01 mM) and TC (0.1∼1.6 mM), borate buffer (10 mM), pH = 8.4, at RT
74	methylene blue (MB)	4-methoxyaniline	(4.58 ± 0.11) × 10^9^	^21^	LFP, DK, excited at 665 nm, monitored at 832 nm, *k*_0_ = 2.4 × 10^4^ s^–1^, N_2_-saturated aqueous solution containing MB^+^ (0.01 mM) and TC (0.1∼1.6 mM), borate buffer (10 mM), pH = 8.4, at RT
75	methylene blue (MB)	4-methylaniline	(4.15 ± 0.05) × 10^9^	^21^	LFP, DK, excited at 665 nm, monitored at 832 nm, *k*_0_ = 2.4 × 10^4^ s^–1^, N_2_-saturated aqueous solution containing MB^+^ (0.01 mM) and TC (0.1∼1.6 mM), borate buffer (10 mM), pH = 8.4, at RT
76	methylene blue (MB)	4-*N*,*N*-dimethylaminoaniline	(4.85 ± 0.16) × 10^9^	^21^	LFP, DK, excited at 665 nm, monitored at 832 nm, *k*_0_ = 2.4 × 10^4^ s^–1^, N_2_-saturated aqueous solution containing MB^+^ (0.01 mM) and TC (0.1∼1.6 mM), borate buffer (10 mM), pH = 8.4, at RT
77	methylene blue (MB)	4-*tert*-butylaniline	(3.27 ± 0.09) × 10^9^	^21^	LFP, DK, excited at 665 nm, monitored at 832 nm, *k*_0_ = 2.4 × 10^4^ s^–1^, N_2_-saturated aqueous solution containing MB^+^ (0.01 mM) and TC (0.1∼1.6 mM), borate buffer (10 mM), pH = 8.4, at RT
78	methylene blue (MB)	aniline	(4.10 ± 0.05) × 10^8^	^21^	LFP, DK, excited at 665 nm, monitored at 832 nm, *k*_0_ = 2.4 × 10^4^ s^–1^, N_2_-saturated aqueous solution containing MB^+^ (0.01 mM) and TC (0.1∼1.6 mM), borate buffer (10 mM), pH = 8.4, at RT
79	methylene blue (MB)	*N*-methylaniline	(4.30 ± 0.17) × 10^9^	^21^	LFP, DK, excited at 665 nm, monitored at 832 nm, *k*_0_ = 2.4 × 10^4^ s^–1^, N_2_-saturated aqueous solution containing MB^+^ (0.01 mM) and TC (0.1 ∼ 1.6 mM), borate buffer (10 mM), pH = 8.4, at RT
80	*N*-methyltryptophan	lipoate	(3.1 ± 0.2) × 10^9^	^126^	LFP, DK, excited at 265 nm, monitored at 440 nm, *k*_0_ = 7.5 × 10^4^ s^–1^, aqueous buffered solution (∼2–10 mM) containing *N*-methyltryptophan (0.15 mM) and TC (∼0.1 mM), pH = 7.5, at RT
81	phenosafranine	indole	0.21 × 10^9^	^127^	LFP, DK, excited at 532 nm, monitored at 785 nm, *k*_0_ = 1.67 × 10^4^ s^–1^, est., Ar-saturated aqueous solution containing phenosafranine and TC (0∼3 mM), Sens concentration: NR, phosphate buffer (1 mM), pH = 7.0
82	phenosafranine	indole-3-acetic acid	4.3 × 10^9^	^127^	LFP, DK, excited at 532 nm, monitored at 785 nm, *k*_0_ = 1.67 × 10^4^ s^–1^, est., Ar-saturated aqueous solution containing phenosafranine and TC (0∼3 mM), Sens concentration: NR, phosphate buffer (1 mM), pH = 7.0
83	phenosafranine	indole-3-butyric acid	6.0 × 10^9^	^127^	LFP, DK, excited at 532 nm, monitored at 785 nm, *k*_0_ = 1.67 × 10^4^ s^–1^, est., Ar-saturated aqueous solution containing phenosafranine and TC (0∼3 mM), Sens concentration: NR, phosphate buffer (1 mM), pH = 7.0
84	phenosafranine	indole-3-carboxylic acid	0.18 × 10^9^	^127^	LFP, DK, excited at 532 nm, monitored at 785 nm, *k*_0_ = 1.67 × 10^4^ s^–1^, est., Ar-saturated aqueous solution containing phenosafranine and TC (0∼3 mM), Sens concentration: NR, phosphate buffer (1 mM), pH = 7.0
85	phenosafranine	indole-3-propionic acid	5.8 × 10^9^	^127^	LFP, DK, excited at 532 nm, monitored at 785 nm, *k*_0_ = 1.67 × 10^4^ s^–1^, est., Ar-saturated aqueous solution containing phenosafranine and TC (0∼3 mM), Sens concentration: NR, phosphate buffer (1 mM), pH = 7.0
86	phenosafranine	tryptophan	0.68 × 10^9^	^127^	LFP, DK, excited at 532 nm, monitored at 785 nm, *k*_0_ = 1.67 × 10^4^ s^–1^, est., Ar-saturated aqueous solution containing phenosafranine and TC (0∼3 mM), Sens concentration: NR, phosphate buffer (1 mM), pH = 7.0
87	pyridine	anthracene	9.5 × 10^8^	^128^	PR, DK, monitored at 310 nm, *k*_0_ = 1.39 × 10^7^ s^–1^, Ar-saturated aqueous solution containing pyridine and TC (0.1∼100 mM), Sens concentration: NR, *T* = 293 K
88	pyridine	biphenyl	1.0 × 10^9^	^128^	PR, DK, monitored at 310 nm, *k*_0_ = 1.39 × 10^7^ s^–1^, Ar-saturated aqueous solution containing pyridine and TC (0.1∼100 mM), Sens concentration: NR, *T* = 293 K
89	pyridine	naphthalene	1.5 × 10^9^	^128^	PR, DK, monitored at 310 nm, *k*_0_ = 1.39 × 10^7^ s^–1^, Ar-saturated aqueous solution containing pyridine and TC (0.1∼100 mM), Sens concentration: NR, *T* = 293 K
90	resazurin (RZ)	2,5-dimethyl-*p*-benzoquinone	5.2 × 10^9^	^129^	LFP, DK, excited at 532 nm, monitored at 825 nm, *k*_0_ = 1.25 × 10^4^ s^–1^ est., Ar-saturated aqueous solution containing RZ and TC (∼0.3 mM), Sens concentration: NR, pH = 10.0, at RT
91	resazurin (RZ)	duroquinone	2.5 × 10^9^	^129^	LFP, DK, excited at 532 nm, monitored at 825 nm, *k*_0_ = 1.25 × 10^4^ s^–1^ est., Ar-saturated aqueous solution containing RZ and TC (∼0.3 mM), Sens concentration: NR, pH = 10.0, at RT
92	resazurin (RZ)	methyl-*p*-benzoquinone	4.5 × 10^9^	^129^	LFP, DK, excited at 532 nm, monitored at 825 nm, *k*_0_ = 1.25 × 10^4^ s^–1^ est., Ar-saturated aqueous solution containing RZ and TC (∼0.3 mM), Sens concentration: NR, pH = 10.0, at RT
93	resazurin (RZ)	*p*-benzoquinone	4.1 × 10^9^	^129^	LFP, DK, excited at 532 nm, monitored at 825 nm, *k*_0_ = 1.25 × 10^4^ s^–1^ est., Ar-saturated aqueous solution containing RZ and TC (∼0.3 mM), Sens concentration: NR, pH = 10.0, at RT
94	resorufin	1-chloro-*p*-benzoquinone	3.0 × 10^9^	^129^	LFP, DK, excited at 532 nm, monitored at 700 nm, Ar-saturated aqueous solution containing resorufin and TC, pH = 10.0, Sens concentration: NR, TC concentration: NR, at RT
95	resorufin	2,5-dimethyl-*p*-benzoquinone	4.9 × 10^9^	^129^	LFP, DK, excited at 532 nm, monitored at 700 nm, Ar-saturated aqueous solution containing resorufin and TC, pH = 10.0, Sens concentration: NR, TC concentration: NR, at RT
96	resorufin	duroquinone	2.8 × 10^9^	^129^	LFP, DK, excited at 532 nm, monitored at 700 nm, Ar-saturated aqueous solution containing resorufin and TC, pH = 10.0, Sens concentration: NR, TC concentration: NR, at RT
97	resorufin	methyl-*p*-benzoquinone	4.1 × 10^9^	^129^	LFP, DK, excited at 532 nm, monitored at 700 nm, Ar-saturated aqueous solution containing resorufin and TC, pH = 10.0, Sens concentration: NR, TC concentration: NR, at RT
98	resorufin	*p*-benzoquinone	2.9 × 10^9^	^129^	LFP, DK, excited at 532 nm, monitored at 700 nm, Ar-saturated aqueous solution containing resorufin and TC, pH = 10.0, Sens concentration: NR, TC concentration: NR, at RT
99	rhodamine 6G (R6G)	ascorbic acid	8.0 × 10^8^	^130^	FP, DK, deoxygenated buffered aqueous solution containing R6G (5.0 × 10^–3^ mM) and TC (∼1.2 × 10^–2^ mM)
100	rhodamine 6G (R6G)	hydroquinone	2.0 × 10^9^	^130^	FP, DK, deoxygenated buffered aqueous solution containing R6G (5.0 × 10^–3^ mM) and TC (∼1.2 × 10^–2^ mM)
101	rhodamine 6G (R6G)	*m*-dinitrobenzene	3.8 × 10^9^	^130^	FP, DK, deoxygenated buffered aqueous solution containing R6G (5.0 × 10^–3^ mM) and TC (∼1.2 × 10^–2^ mM)
102	rhodamine 6G (R6G)	*p*-benzoquinone	2.5 × 10^9^	^130^	FP, DK, deoxygenated buffered aqueous solution containing R6G (5.0 × 10^–3^ mM) and TC (∼1.2 × 10^–2^ mM)
103	rhodamine 6G (R6G)	*p*-phenylenediamine	1.0 × 10^9^	^130^	FP, DK, deoxygenated buffered aqueous solution containing R6G (5.0 × 10^–3^ mM) and TC (∼1.2 × 10^–2^ mM)
104	rose bengal (RB)	gadusolate	(2.0 ± 0.1) × 10^8^	^131^	LFP, DK, excited at 532 nm, monitored at 590 nm, *k*_0_ = 2 × 10^4^ s^–1^ est., aqueous solution containing RB (4 × 10^–3^ mM) and TC (0.6 mM), pH = 7.0
105	tryptophan (Trp)	lipoate	(3.6 ± 0.4) × 10^9^	^126^	LFP, DK, excited at 265 nm, monitored at 440 nm, *k*_0_ = 7.0 × 10^4^ s^–1^, aqueous buffered solution (∼2–10 mM) containing Trp (0.15 mM) and TC (∼0.1 mM), pH = 7.3, at RT

a The dataset was compiled from peer-reviewed studies reporting explicit kinetic measurements under defined experimental conditions. Abbreviations used in this table: DK, direct kinetics; FP, flash photolysis; *k*_0_ is the first-order decay rate constant in the absence of TC; NR denotes information not reported; RT represents room temperature (298 K unless otherwise specified).

Beyond organic TCs, LFP has also been used to investigate quenching of ^3^Sens* by inorganic species.^37,38^ Available kinetic data are concentrated mainly on halide ions and carbonate. Halide ions quench triplet-state aromatic ketones with *k*_q_ values ranging from approximately 10^9^ M^–1^ s^–1^ for I^–^ to below 10^5^ M^–1^ s^–1^ for Cl^–^.^39^ CO_3_^2–^ can also quench organic ^3^Sens*. Direct LFP measurements for five Sen yielded *k*_q_ values of up to 3.0 × 10^7^ M^–1^ s^–1^ and confirmed the formation of CO_3_^•–^.^40^ The competitive importance of an inorganic quencher in environmental waters depends not only on its *k*_q_ value, but also on its concentration, as represented by the pseudo-first-order loss term *k*_q_[ion]. Consequently, abundant inorganic ions may contribute appreciably to ^3^Sens* decay even when their *k*_q_ values are lower than those of trace organic TCs. Such competitive quenching decreases the fraction of ^3^Sens* available for pollutant transformation and, for reactive halides, may also initiate secondary halogen-radical chemistry. These effects are relevant not only to marine aerosols and sea-salt particles, but also to surface waters containing elevated concentrations of inorganic ions. In contrast, direct *k*_q_ data for nitrate, phosphate, and sulfate remain limited, including in atmospheric aqueous-phase systems, highlighting the need for systematic kinetic measurements.

Despite its versatility, LFP has important limitations. It becomes less reliable for slow quenching reactions, where *k*_q_ values fall below 10^6^ M^–1^ s^–1^.^41^ High Sens concentrations or Sens with strong molar absorption coefficients can trigger undesired second-order processes such as triplet–triplet annihilation (TTA),^42^ particularly at high excitation intensities. Additionally, systems lacking suitable chromophores in ^3^Sens* for direct detection often require competition kinetics with reference compounds.^43^ While effective, this approach increases the likelihood of side reactions and complicates data interpretation.

### 2.2 Pulse Radiolysis

PR is a powerful technique for probing excited states, providing detailed insights into the kinetics and mechanisms of quenching reactions.^44^ Unlike LFP, which uses optical photons from pulsed lasers, PR employs high-energy electron pulses (in the MeV range) delivered by a linear accelerator to initiate excitation. This higher energy compared to laser photons (in eV range) enables generation of a broader range of reactive species, including solvated electrons (e^-^_aq_), radicals, and excited states.

In dilute solutions, PR primarily generates reactive species through the solvent, as radiation absorption occurs proportionally to the mass fraction of components present. Energy initially deposited in the solvent transfers to the solute via energy transfer or ion recombination processes.^45^ The resulting excited states can be monitored by optical spectroscopy, electrical conductivity, or electron spin resonance (ESR), depending on the species of interest.

For monitoring transient signals, optical spectroscopy is frequently used to track ^3^Sens* behavior by measuring light absorption changes at specific wavelengths.^46^ Electrical conductivity measurements detect changes in current flow, indirectly monitoring charge-transfer process and ion generation. ESR detects signals from unpaired electrons using specific microwave frequencies, enabling product quantification.^47^ However, ESR has limitations. Signal intensity varies with the dielectric constant of the system, and prolonged reactions can cause microwave energy accumulation in the measurement cavity, potentially distorting ^3^Sens* detection.

PR has proven valuable for investigating quenching kinetics of ^3^Sens* by various TCs, especially in systems where LFP is limited by the requirement that Sens must exhibit sufficient absorption at the excitation wavelength. In contrast to photon-based excitation, PR generates excited states through high-energy electron irradiation and therefore does not rely on the optical absorption properties of the Sens. This feature enables studies of weakly absorbing systems or systems that are not readily accessible by direct optical excitation. Several studies have used PR to examine triplet aromatic ketone quenching by phenolic compounds, with transient absorption signals allowing quantification of *k*_q_ and identification of intermediate species.^20,46,48^

A significant limitation of PR stems from its non-selective excitation mechanism. The high-energy (typically 7 MeV) electrons generate multiple reactive species simultaneously, including *e*_aq_^–^,^•^OH, hydrogen atoms (H^•^, the protonated form of *e*_aq_^–^), and ^3^Sen*.^45^ These species can interfere with reactions of ^3^Sens* and TCs, complicating *k*_q_ determination. LFP may also generate unwanted photoproducts or radical intermediates, but it is generally less affected by this limitation than PR because laser excitation is wavelength-selective. In contrast, PR relies on non-selective high-energy electron irradiation and therefore produces a broader suite of primary reactive species. Reliable *k*_q_ values can be determined only when the ^3^Sens* signal is both kinetically and spectrally isolated. This generally requires monitoring a characteristic absorption band of ^3^Sens*, selecting a time window in which ^3^Sens* decay dominates, and using scavengers, control experiments, or multiwavelength analysis to distinguish ^3^Sens* decay from other transient processes. TC-derived radicals may also form during quenching reactions. If their absorption overlaps with that of ^3^Sens*, additional spectral or kinetic controls are needed to ensure that the extracted *k*_q_ reflects ^3^Sens* decay rather than secondary radical chemistry.

Solvent polarity significantly affects reactive species generation in PR experiments. Nonpolar solvents such as hexane and benzene favor ^3^Sens* production through ion recombination while yielding relatively few radical ions. Conversely, polar solvents like water and short-chain alcohols rapidly solvate and stabilize initial ions, slowing ion recombination and producing high yields of radical ions but low yields of ^3^Sens*.^49^ This solvent dependence is specific to PR and differs from other techniques like LFP, where direct solute excitation minimizes solvent-mediated interferences. Consequently, ^3^Sens* studies are generally more effective in non-polar solvents using PR, while polar solvents better facilitate examination of radicals and radical ions.^31^

While aqueous systems remain central to water-relevant quenching kinetics studies, researchers often employ non-aqueous and mixed-solvent systems for mechanistic investigations. These alternative media provide better control over solubility, polarity, and reactive-species composition. For example, Canonica et al. used acetonitrile–water mixtures to study aromatic ketones and humic substance quenching kinetics, addressing the compounds’ limited water solubility.^50^ Additionally, organic solvents like benzene and cyclohexane have been used to isolate ^3^Sens* from water-derived reactive species (*e.g*., ^•^OH and *e*_aq_^–^), enabling clearer interpretation of intrinsic quenching kinetics.^51,52^

### 2.3 Time-Resolved Chemically Induced Dynamic Nuclear Polarization

TR-CIDNP combines pulsed laser excitation with Fourier-transform NMR detection to indirectly monitor short-lived radical intermediates in photochemical reactions.^53^ In this method, laser excitation promotes a Sens molecule to its triplet state, which then undergoes photoinduced electron transfer (PET), hydrogen atom transfer (HAT), or proton-coupled electron transfer (PCET) with a TC. This interaction generates spin-correlated radicals, typically TC^•+^ and Sens^•–^, which form a radical pair (RP) within a solvent cage. During diffusion or recombination, hyperfine interactions between unpaired electrons and nearby nuclear spins create non-Boltzmann nuclear polarization, detectable as enhanced or inverted NMR signals (Figure 4). The CIDNP signal intensity is proportional to the yield of spin-correlated radical pairs, enabling specific assessment of radical generation efficiency. TR-CIDNP provides a valuable alternative for determining *k*_q_ when optical methods are limited by spectral overlap or insufficient chromophore absorbance.^54^

**Figure 4:**
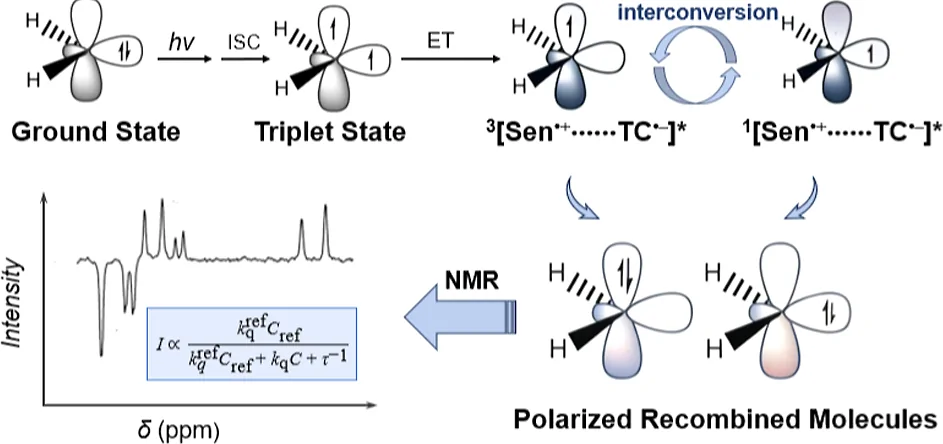
Schematic illustration of TR-CIDNP signal generation. Photoexcitation forms a radical pair whose singlet–triplet interconversion induces nuclear spin polarization, resulting in enhanced absorption or emission signals observed in the NMR spectrum.

The CIDNP intensity follows Stern–Volmer kinetics relative to TC concentration (eq 1).^55^ This relationship enables determination of unknown *k*_q_ values by monitoring the CIDNP signal intensities of a transient product formed from a reference compound (RC)

2 $$k_{q}=k_{q}^{RC}\left(\frac{I_{0}}{I}-1\right)\frac{C_{RC}}{C_{TC}}$$

where *k*_q_^RC^ represents the *k*_q_ value for the reaction between RC and ^3^Sens*, *I*_0_ is the CIDNP intensity of RC without TC, *I* is the CIDNP intensity with TC present. When TC and RC concentrations are equal, eq 2 simplifies to

3 $$k_{q}=k_{q}^{RC}\left(\frac{I_{0}}{I}-1\right)$$

To illustrate TR-CIDNP application in quenching kinetics, consider this example: The technique was used to study the quenching of triplet 3,3′,4,4′-tetracarboxy benzophenone and 1-methyl histidine, using *N*-acetyl histidine as the RC.^56^ Using an NMR detector and a 308 nm excimer laser, researchers determined *k*_q_ as 1.1 × 10^9^ M^–1^ s^–1^, with an *I*/*I*_0_ ratio of 0.22 and a *k*_q_^RC^ value of 3.0 × 10^9^ M^–1^ s^–1^.

This approach requires that the RC produces a strong, well-resolved CIDNP signal without spectral overlap with ^3^Sens* or TC signals. Common RCs include amino acid derivatives like methyl histidine and β-alanyl-1-methyl histidine, which provide high CIDNP sensitivity with minimal interference. The prevalence of amino acid–based RCs reflects the widespread use of TR-CIDNP in investigating photoinduced mechanisms in biological environments, particularly with peptides and proteins. This technique may also apply to probing the excited-state kinetics of DOM in aquatic environments, as DOM often contains amino acid-like moieties capable of similar radical polarization processes.

Note that TR-CIDNP is limited to quenching reactions proceeding via radical pathways, as CIDNP signals originate from the spin-selective recombination of radical pairs, dependent on electron spin multiplicity.^56^ Additionally, as shown in eq 3, the accuracy of the determined *k*_q_ value depends directly on the reliability of *k*_q_^RC^, which constrains the general applicability.

## 3 Determinants of Quenching Efficiency: Structural and Environmental Controls

The *k*_q_ dataset in Table 2 was compiled from studies that reported explicit kinetic measurements for quenching between identifiable ^3^Sens* and organic TCs. Entries were included only when the Sens, TC, kinetic method, and reported *k*_q_ value could be clearly assigned. Studies lacking sufficient kinetic information, lacking clearly defined ^3^Sens*–TC pairs, or reporting only qualitative quenching behavior were not included. The resulting dataset covers different Sens classes, TC structures, pH values, temperatures, ionic strengths, and reactant concentrations.

The *k*_q_ values (*n* = 105) for various TCs reacting with ^3^Sens* in aqueous solutions are summarized in Table 2. These values were mainly determined by time-resolved techniques such as LFP, PR, and TR-CIDNP, typically under dilute and optically transparent aqueous conditions. Quenching efficiency depends on two main factors: (1) molecular characteristics of TCs, such as functional groups, conjugation, and substituent positions; and (2) environmental conditions including pH, temperature, and solvent composition. However, the current dataset is heavily biased toward idealized aqueous systems, whereas actual environmental matrices (cloudwaters, aerosols, and surface waters) exhibit substantial variability in parameters like ionic strength, polarity, and viscosity. For context, the ionic strength in dilute cloud, fog, and rain droplets is at least 10^–4^ M, while deliquescent aerosol particles can reach near-molar levels.^57^ Although high-ionic-strength environments are common in nature, particularly in marine aerosols and hypersaline lakes, their effect on *k*_q_ values remains poorly understood due to limited systematic studies under such conditions. Addressing this knowledge gap is critical for improving the transferability of laboratory kinetic data to real-world environmental systems.

### 3.1 Molecular Features of Target Compounds

#### 3.1.1 Electron Donating/Electron Withdrawing Groups

Environmentally relevant TCs refer to organic pollutants of environmental concern in environmental waters and atmospheric aqueous phases, including aromatic amines, phenols, polyphenols, aromatic carboxylic acids, nitrogen-containing heterocycles, PAHs and their derivatives, and halogenated aromatics. The functional groups on these TCs significantly influence their interaction with ^3^Sens*, thereby affecting quenching kinetics.

Electron-donating groups (EDGs) such as hydroxyl, alkyl, and amino groups enhance the interactions, typically resulting in high *k*_q_ values. For the quenching of triplet-state methylene blue by meta-substituted aniline derivatives, anilines bearing hydroxy, alkyl, or alkoxy substituents exhibited *k*_q_ values on the order of 10^9^ M^–1^ s^–1^, whereas 3-fluoroaniline and 3-chloroaniline showed much lower *k*_q_ values of approximately 10^7^ M^–1^ s^–1^. Phenolic compounds show similar trends, with hydroxyl substitution facilitating rapid quenching and EWGs slowing the process. While EDG/EWG substitution patterns clearly control electron-transfer reactivity with ^3^Sens*, systematic assessments across broader compound classes remain limited.

Conjugation effects further illustrate how structural features govern ^3^Sens* quenching. Extended π-conjugation in dienes, aromatics, and heteroaromatics, generally enhances electron transfer by raising the highest occupied molecular orbital (HOMO) energy and improving electron-donating ability, leading to higher *k*_q_ values. Conjugated diolefins typically quench ^3^Sens* more efficiently than their non-conjugated analogues,^58^ while additional double bonds or fused rings in aromatics also increase quenching efficiency. Heteroaromatic compounds exhibit similar behavior, with conjugation to electron-donating substituents further accelerating electron transfer.

#### 3.1.2 Substituent Position

Substituent position affects TC reactivity with ^3^Sens* by modulating both electronic structure and steric accessibility. Steric effects alter molecular approach geometries, reduce frontier orbital overlap, and decrease productive diffusion encounters, directly influencing observed *k*_q_ values. Methoxybenzene isomers clearly demonstrate this effect: 1,2,4-trimethoxybenzene exhibits a *k*_q_ near 10^9^ M^–1^ s^–1^, significantly higher than both 1,2,3- (10^8^ M^–1^ s^–1^) and 1,3,5-isomers (10^7^ M^–1^ s^–1^). This variation stems from combined electronic factors and reduced steric hindrance, allowing closer interaction with triplet-state benzophenone.^59^ Similar trends are observed across other molecular classes. For example, bulky ortho-substituents on pyrene derivatives suppress quenching efficiency by tertiary aliphatic amines,^60^ while ortho-substituted aromatic amines consistently quench triplet states less efficiently than their para- or meta-substituted analogues.^61^

Despite this evidence for steric effects in triplet-state quenching, comprehensive and quantitative evaluations remain limited. Most data come from case-specific comparisons, making mechanistic trend generalization difficult. Additionally, the inherent coupling between steric and electronic influences often obscures their individual contribution to quenching kinetics. Future research should combine controlled experimental series of positional and substituent analogues with quantum chemical analyses to better separate steric and electronic effects. Such integrated approaches would provide a predictive framework for understanding how molecular architecture governs *k*_q_ values across diverse ^3^Sens*–TC systems.

Overall, thermodynamic descriptors such as *E*_T_ and redox driving force define the feasibility of a quenching pathway, whereas molecular interactions, including encounter geometry, steric accessibility, donor–acceptor distance, orbital overlap, and electronic coupling, govern the probability of productive quenching and thereby modulate *k*_q_. This distinction helps explain why ^3^Sens*–TC pairs with similar energetic driving forces may still exhibit different *k*_q_ values. The compiled *k*_q_ dataset reveals several structure-reactivity trends. Electron-rich TCs, including phenols, anilines, and indole derivatives, generally show higher *k*_q_ values toward electron-deficient ^3^Sens*, consistent with electron-transfer or proton-coupled electron-transfer pathways. EWG substituents often decrease *k*_q_ by reducing the electron-donating ability of TCs, whereas deprotonation can increase *k*_q_ by increasing electron density and changing hydrogen-bonding interactions. Substituent position and steric hindrance further regulate encounter efficiency between ^3^Sens* and TCs. These trends should be interpreted with caution because the reported values were obtained using different Sens, methods, pH values, and solution conditions. Nevertheless, the dataset shows that *k*_q_ is controlled by coupled electronic, steric, and environmental factors rather than by a single molecular descriptor. The curated *k*_q_ dataset may also provide a useful starting point for future quantitative structure–reactivity relationships or machine-learning models. However, such models should be developed cautiously because reliable prediction requires high-quality kinetic data, consistent experimental conditions, and sufficient coverage of molecular structures.

### 3.2 Environmental Parameters

#### 3.2.1 pH

Environmental waters exhibit a wide pH range due to various natural and anthropogenic factors. Large freshwater bodies typically maintain near-neutral values (pH 6.0–8.0),^62^ while cloud and fog droplets are mildly acidic (pH 3.0–5.0) due to dissolved CO_2_ and organic acids.^63,64^ More extreme acidity (pH 1.0–3.0) occurs in particulate matter, like soot and mineral dust carrying adsorbed HNO_3_ or H_2_SO_4_.^63^

The protonation states of both ^3^Sens* and TCs vary across these pH conditions, significantly affecting their redox potentials, absorption characteristics, and quenching reactivity. These effects depend primarily on the p*K*_a_ values of their functional groups. While most studies in Table 2 report *k*_q_ values at a single pH (typically 7.0–8.0), some have systematically investigated pH effects on quenching.^65,66^

The pH-dependence of *k*_q_ is often nonlinear, reflecting changes in protonation state and reaction mechanisms. For example, the quenching of triplet 2,2′-dipyridyl (^3^DP*, p*K*_a_ = 5.8) by guanosine-5′-monophosphate (GMP, p*K*_a1_ = 2.4, p*K*_a2_ = 9.4) shows distinct mechanistic regions:^67^ between pH 2.4–5.8, protonated ^3^DPH*^+^ rapidly reacts with GMP (*k*_q_ = 2.7 × 10^9^ M^–1^ s^–1^) via electron transfer. From pH 5.8–9.4, partial deprotonation leads to slower HAT (*k*_q_ = 1.6 × 10^8^ M^–1^ s^–1^). Above pH 9.4, fully deprotonated GMP restores faster reactivity (*k*_q_ = 1.1 × 10^9^ M^–1^ s^–1^).

Similar pH effects appear in atmospheric systems. The *k*_q_ values for 3-methoxyacetophenone triplet (^3^3-MAP*) increased nearly 100-fold, from 1.0 × 10^7^ M^–1^ s^–1^ at pH 2.0 to 9.8 × 10^8^ M^–1^ s^–1^ at pH 9.0.^68^ This behavior often reflects fundamental changes in redox properties—phenolate (deprotonated phenol) is more readily oxidized than phenol (p*K*_a_ = 9.9), due to its lower one-electron reduction potential (Figure 5).^69,70^ Beyond changes in protonation state and redox potential, pH can alter hydrogen-bonding interactions within ^3^Sens*–TC encounter complexes. Hydrogen bonding may preorganize the electron-donor and proton-transfer sites, modify electronic coupling, and facilitate PCET or, in suitable systems, HAT. Accordingly, pH-dependent changes in *k*_q_ may reflect not only changes in molecular speciation and redox thermodynamics, but also changes in hydrogen-bonding interactions and the relative contributions of PET, PCET, and HAT. However, *k*_q_ trends alone are insufficient for pathway assignment; transient spectral evidence, kinetic isotope effects, thermodynamic analysis, and product identification are also required.

**Figure 5:**
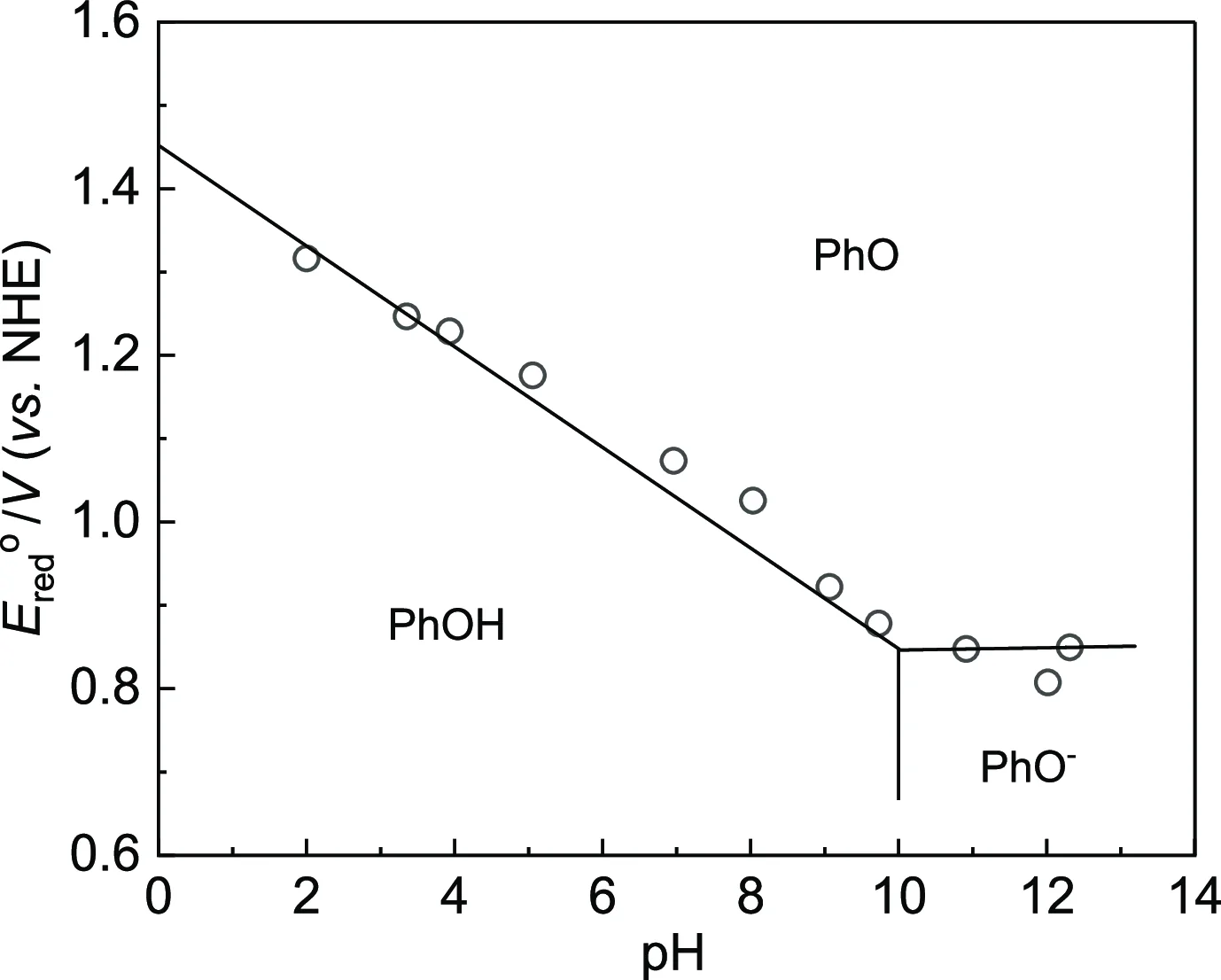
One-electron reduction potentials (*E*_red_^o^) of phenol (PhOH) vary with pH. Error bars of ±19 mV are within the points. Reproduced from ref 69. Copyright 1999 American Chemical Society.

For chromophoric dissolved organic matter (CDOM), the predominant natural Sens in surface waters, pH significantly affects photobleaching efficiency.^71^ Suwannee River humic acid (SRHA) shows a 51% increase in photobleaching at 330 nm as pH rises from 3.7 to 9.3,^72^ attributed to chromophore expansion that increases light absorption.^72,73^

pH also controls competitive quenching processes through its effect on inorganic carbon speciation. In lakes, increasing pH shifts the carbonate equilibrium toward CO_3_^2–^, which scavenges ^•^OH and promotes CO_3_^•–^ formation, thereby altering dominant photochemical pathways.^74^ This effect arises from pH-dependent carbonate speciation rather than from a general increase in ionic strength. Other inorganic ions, such as Cl^–^ and Br^–^, may also influence ^3^Sens*-mediated reactions, but their effects are not primarily governed by pH-dependent speciation. Instead, they may contribute through direct quenching of certain ^3^Sens*, ion-specific interactions, or subsequent formation of reactive halogen species. Despite its apparent significance, the influence of pH and inorganic ion composition on ^3^Sens*-mediated processes remains under-investigated and warrants systematic study.

#### 3.2.2 Solvent Properties and Ionic Strength

Although many environmental photochemical studies focus on the aqueous phase, understanding how solvent properties such as polarity (μ, D), viscosity (η, cP) and ionic strength influence *k*_q_ remains crucial for interpreting and generalizing reactivity trends,^75^ especially under non-ideal or atmospheric conditions. Environmental systems vary widely in solvent properties: high salinity in marine and brine waters increases ionic strength; DOM-rich or microgel-like phases raise viscosity; and organic-coated or interfacial regions reduce polarity. These physicochemical differences can substantially alter both the diffusion behavior of reactive species and the electronic coupling between ^3^Sens* and TCs.

Solvent polarity and viscosity govern solvent–solute interactions and thus influence *k*_q_ values. Lower polarity weakens electron-transfer processes by reducing stabilization of charge-separated intermediates. This effect is demonstrated by studies of triplet fluorenone (^3^FL*) quenching by amines in solvents of different polarity. For the non-aromatic diamine diazabicyclo-octane, *k*_q_ decreases by over an order of magnitude when the solvent changes from acetonitrile (3.92 D) to toluene (0.37 D),^76^ reflecting strong dependence on solvent polarity. In contrast, the aromatic amine dimethyl-*p*-toluidine exhibits only a modest decrease in *k*_q_ under the same conditions.^77^ Its π-delocalized HOMO enables efficient orbital overlap with ^3^FL* and stabilizes associated charge-transfer states, making the reaction markedly less sensitive to solvent polarity.

*k*_q_ values of EnT vary with solvent viscosity, and are thus not always comparable to diffusion controlled rates (*k*_D_).^78^ For example, the quenching efficiency of conjugated dienes on triplet-state valerophenone was measured in different solvents.^79^ The *k*_q_ value is 1.8 × 10^8^ M^–1^ s^–1^ in hexane (0.33 cP), with *k*_D_ being 4.9 × 10^8^ M^–1^ s^–1^, while in cyclooctane (2.16 cP), the *k*_q_ value is 7.0 × 10^7^ M^–1^ s^–1^ and *k*_D_ is 7.5 × 10^7^ M^–1^ s^–1^. In the less viscous solvent hexane, the *k*_q_ value for EnT is slower than *k*_D_, indicating that the ^3^Sens* and TC tend to diffuse apart before energy transfer occurs, reducing its probability. However, viscosity in environmental aqueous systems typically ranges narrowly (0.8–2.0 cP), so such moderate variations rarely cause significant deviations in *k*_q_.

Ionic strength is a key parameter in natural waters, ranging from below 1 mM in precipitation and cloud water to near-molar levels in seawater, brines, or aerosol droplets.^57,80^ Previous studies indicate that increasing ionic strength decreases *k*_q_, likely due to two intertwined mechanisms: (1) screening of electrostatic interactions between charged or polarizable species, reducing encounter probability; and (2) increased activation free energy from solvent reorganization.^26^ This effect is exemplified by the quenching of excited quinine by Cl^–^,^81^ where the Stern–Volmer constant (*k*_SV_, M^–1^)^12,82^ is given by

4 $$\frac{\tau_{0}}{\tau }=1+k_{SV}\left[TC\right]$$

τ_0_ (s) and τ (s) are the lifetimes of excited Sens (quinine in this case) without and with Cl^–^, respectively; and *k*_SV_ equals the product of *k*_q_ and τ_0_. *k*_SV_ values for Cl^–^ mediated quenching of quinine fluorescence decreased from 133 to 75 M^–1^, as the SO_4_^2–^ concentration rose from 0.1 to 1.0 M. Acid concentration had negligible effects on excited quinine lifetime, confirming that changes in *k*_SV_ mainly reflect variations in *k*_q_. Calculated *k*_q_ values were 7.8 × 10^9^ and 3.8 × 10^9^ M^–1^ s^–1^ at SO_4_^2–^ concentrations of 0.1 and 1.0 M, respectively, indicating the inverse correlation between *k*_q_ and ionic strength. Control experiments confirmed this reduction arises from ionic strength, not pH.^81^ These findings suggest that salinity gradients in natural environments could systematically bias kinetic measurements if not properly accounted for.

#### 3.2.3 Temperature

Temperature is a critical but often underexplored factor in ^3^Sens* quenching in natural environments. Liquid natural waters typically range from below −2 °C for seawater in winter to over 30 °C in lakes and shallow streams, with seasonal fluctuations often exceeding 20 °C. Atmospheric systems such as cloud droplets and aerosols experience even broader ranges, including sub-zero conditions. In snow and ice phases, the solid-state environment constrains molecular mobility and modifies solvation, fundamentally altering interactions between ^3^Sens* and TCs.^83^

Increasing temperature generally enhances molecular motion, raising collision frequency and energy, which often increases *k*_q_. The magnitude of this effect depends on whether quenching is diffusion- or reaction-controlled. In diffusion-controlled systems, temperature affects the diffusion coefficient (per Stokes–Einstein relation) and thus *k*_D_.^84^ In reaction-controlled systems, temperature sensitivity depends on the activation energy (*E*_a_) of the electron transfer, hydrogen atom transfer, or energy transfer steps, typically following Arrhenius-type behavior. Kowalska-Baron et al. studied quenching of triplet-state indole by iodide over 5.0–45 °C.^42^ Without iodide, phosphorescence lifetimes remained nearly constant, indicating no diffusion control (quenching limited by molecular encounters). In the presence of iodide, quenching likely proceeds via an exciplex intermediate (indole as Sens, and iodide as TC)

5 Sens*3+TC⇌k−dkd[Sen−TC]*→kpproducts

The *k*_q_ for quenching of triplet indole by iodide was calculated as

6 $$k_{q}=\frac{k_{d}\times k_{p}}{k_{-d}+k_{p}}$$

where *k*_d_ is the exciplex formation rate constant, *k*_–d_ and *k*_p_ are unimolecular rate constants for exciplex dissociation to reactants and products, respectively. It should be noted that eq 5 describes an encounter-complex framework for this indole–iodide system and should not be regarded as a universal mechanism for all ^3^Sens*–TC reactions. Other ^3^Sens*–TC systems may follow different quenching mechanisms depending on the molecular properties of Sens and TC. As shown in Figure 6, *k*_q_ increased with temperature, suggesting diffusion-control. Higher temperature increases iodide mobility and collision frequency with excited indole, enhancing quenching efficiency. However, excessively high temperatures may accelerate thermal deactivation, causing excited states to lose energy non-radiatively and reducing quenching efficiency.^85^

**Figure 6:**
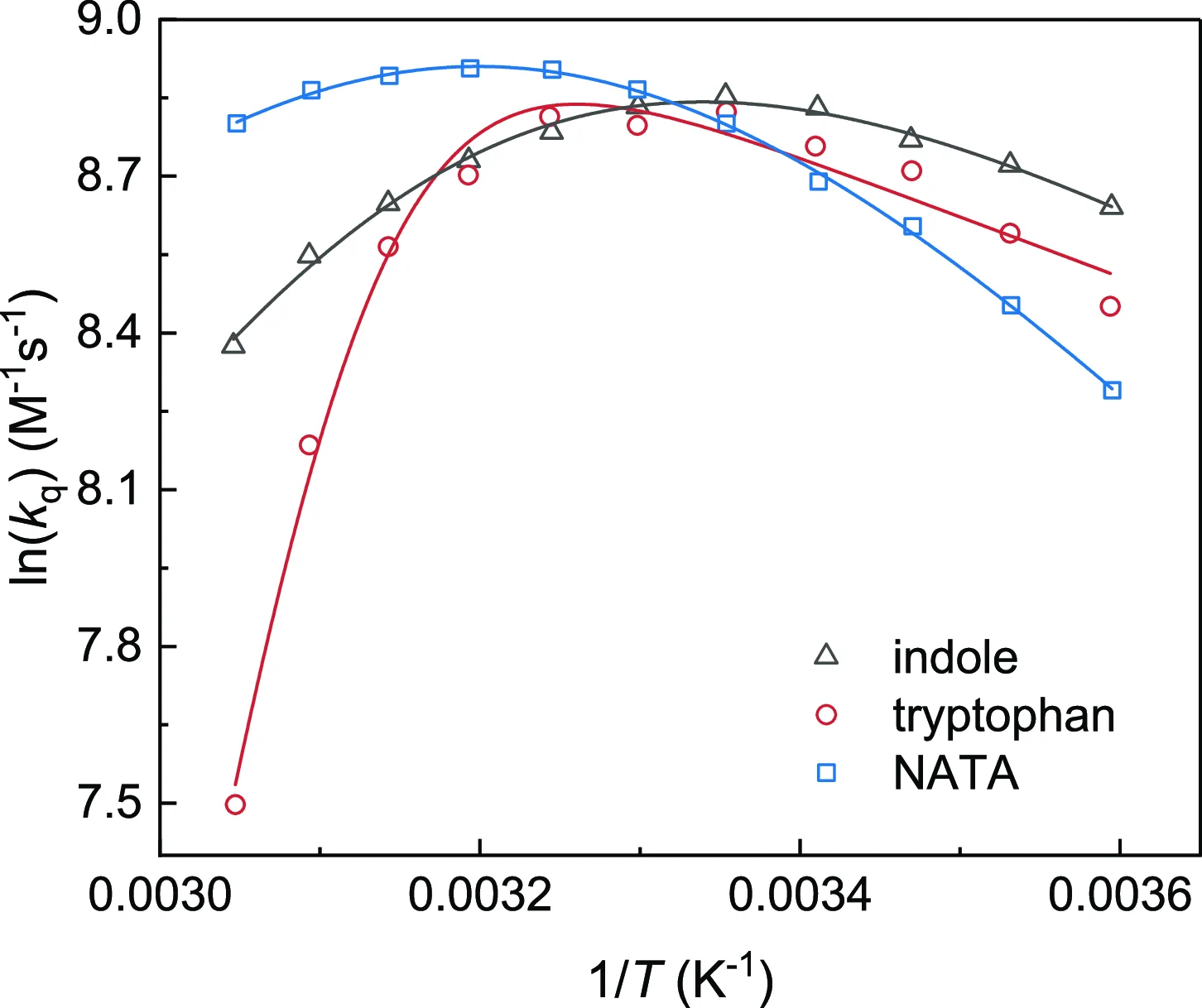
Arrhenius behavior of *k*_q_ for the triplet-state quenching of indole, tryptophan and *N*-acetyl-l-tryptophanamide by iodide in aqueous solution. Reproduced from ref 42. Copyright 2013 Elsevier.

Temperature dependence of quenching reactions varies with the TCs. For example, quenching of triplet 3-methylindole by three TCs (*N*-acetylglycinamide, ethyl acetate, and gadolinium chloride) over 5.0–50 °C showed distinct behaviors: ethyl acetate exhibited no temperature dependence, while gadolinium chloride showed strong sensitivity.^86^ These differences likely reflect varying activation energies, with more temperature-sensitive reactions having higher barriers. Similar temperature-dependent behaviors occur in other systems, such as quenching of aromatic hydrocarbon triplets by oxygen^87^ and carbonyl triplets amines.^88^ These findings indicate that temperature effects on *k*_q_ are not uniform but instead depend strongly on the structural features of the quenching partner.

Despite these insights, systematic studies of temperature effects in environmentally relevant systems remain scarce. Investigations into temperature impact on photochemistry under extremes, such as saline lakes, acidic fog, or cold marine aerosols, are largely lacking. Low temperatures slow diffusion, altering reactivity,^89^ while solid-phase photochemistry in ice remains largely unexplored. Future research integrating temperature with other parameters like pH and ionic strength, especially in cryospheric and atmospheric models is essential to improve mechanistic understanding and environmental relevance.

## 4 Mechanistic Pathways of ^3^Sens*-Mediated Quenching Reactions

Understanding quenching mechanisms is essential to elucidate the principles governing ^3^Sens* reactivity and the factors that modulate *k*_q_ values. The two primary mechanisms are EnT, which redistributes excited state energy via molecular interactions, and redox reactions involving various forms of electron transfer, such as photoinduced electron transfer (PET) and proton-coupled electron transfer (PCET) (Figure 7). Here, PCET is used as a broad category that includes multiple coupled proton–electron transfer modes, with HAT representing one concerted limiting case rather than the only form of PCET.

**Figure 7:**
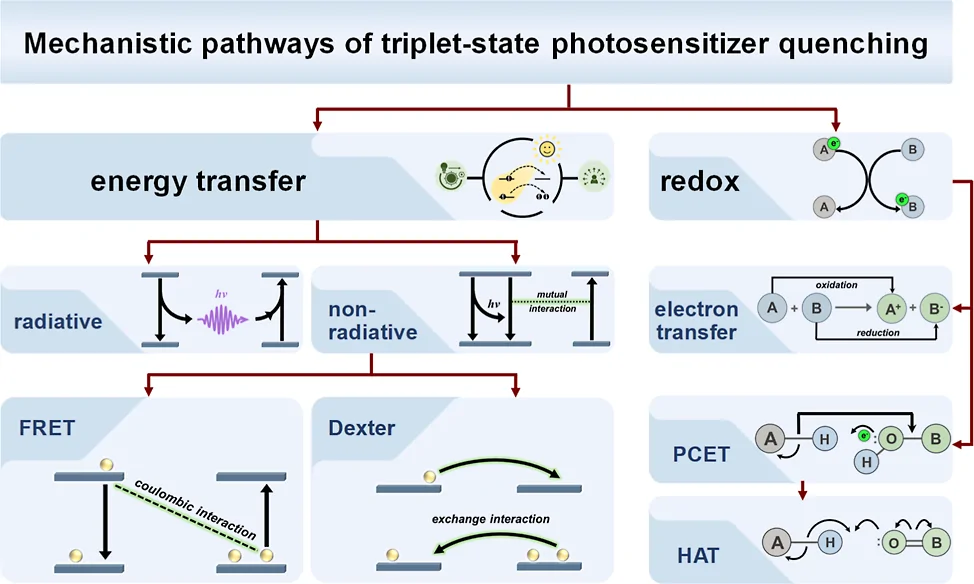
Mechanistic pathways of quenching reactions involving excited triplet-state photosensitizers. Quenching is classified into energy-transfer pathways (radiative energy transfer and non-radiative energy transfer, including Förster resonance energy transfer FRET and dexter energy transfer) and redox pathways (electron transfer, proton-coupled electron transfer PCET, with HAT shown as a concerted PCET pathway).

### 4.1 Energy Transfer Mechanisms

As outlined earlier in the Jablonski diagram (Figure 1), Sens excitation leads to formation of its triplet excited state, which can relax through radiative or non-radiative pathways. Radiative EnT involves a photon emission by ^3^Sens* followed by absorption by TC, also called trivial EnT.^90^ This section focuses on non-radiative EnT, where ^3^Sens* transfers excitation energy directly to TCs without photon emission.^91^ This direct transfer promotes the TC to a reactive excited state, initiating chemical transformations and making non-radiative EnT a key pathway for light-driven degradation of organic pollutants in natural waters and atmospheric aerosols. The process proceeds via two distinct mechanisms governed by fundamental electron–electron interactions: Förster resonance energy transfer (FRET) and Dexter EnT.

FRET is a long-range mechanism involving classical dipole–dipole (Coulombic) interactions, transferring energy via a transmitter-antenna effect.^92^ Oscillations in ^3^Sens* induce a dipole that triggers corresponding oscillations in ground-state TCs via charge repulsion in the electromagnetic field. This resonant interaction between ^3^Sens* and TC results in the relaxation of ^3^Sens* and the simultaneous excitation of TC. FRET between ^3^Sens* and TC proceeds as triplet–singlet energy transfer (^3^Sens* + TC → Sens + ^1^TC*)^93^ and occurs effectively within a distance range of 1–10 nm between ^3^Sens* and TC. The rate constant for FRET (*k*_FRET,_ s^–1^), is given by^19^

7 $$k_{FRET}=\frac{1}{\tau_{0}}\times \left(\frac{R_{0}}{R}\right)6$$

where *R* is the distance between ^3^Sens* and TC, τ_0_ is the lifetime of ^3^Sens* in the absence of TC and *R*_0_ is the critical Förster radius, *i.e*. the distance at which the energy transfer efficiency is 50%.^12,94^ FRET efficiency depends on spectral overlap between ^3^Sens* emission and TC absorption,^95^ as well as on the ^3^Sens* quantum yield, donor–acceptor separation, and the relative orientation of their transition dipoles.^96^

Though FRET reports in environmental studies are scarce, an illustrative case from analytical chemistry is the acridine orange (AO)–rhodamine B (RhB) pair, used to detect vitamin B_12_ in aqueous solution.^97^ In this system, AO is excited and subsequently transfers its energy non-radiatively to RhB through FRET, leading to quenched AO fluorescence and enhanced RhB emission. Vitamin B_12_, which absorbs near the RhB emission band, further quenches the sensitized fluorescence, enabling sensitive detection. This example demonstrates how FRET principles can be harnessed to design highly sensitive fluorescence assays. Similar studies provide methodological inspiration for the future development of FRET-based probes aimed at pollutant monitoring and environmental diagnostics.^98,99^

Dexter EnT is a primary mechanism by which ^3^Sens* transfers energy to generate an excited triplet TC (^3^TC*). It involves simultaneous electron exchange: ^3^Sens* donates an electron to the lowest unoccupied molecular orbital (LUMO) of the TC, while receiving one from the HOMO of the TC. This two-electron exchange requires orbital overlap achievable only at distances typically less than 1 nm. In environmental photochemistry, Dexter EnT occurs mainly via two pathways: triplet–triplet energy transfer, the predominant route for TC triplet excitation (eq 8); and energy transfer to molecular oxygen, sensitizing singlet oxygen formation (eq 9).^93^

8 Sens*3+TC→Sens+T3C*

Sens*+3O23→Sens+O21 9

The Dexter EnT rate constant (*k*_Dexter_, s^–1^) is expressed as

10 $$k_{Dexter}=K\times J\times e^{-2R/L}$$

Here, *K* relates to orbital ^3^Sens*–TC interactions and their orientation dependence; *J* is the spectral overlap integral (M^–1^ cm^–1^); and *L* is the sum of their van der Waals radii.^11^

Dexter-type triplet–triplet energy transfer occurs broadly across diverse photochemical environmental systems (eq 8). Bimolecular energy transfer quenching of triplet states of a series of 2,2′-bipyridine complexes of Os­(II) by anthracene and 2,3-benzanthracene was investigated.^100^ The measured *k*_q_ values range from 6.3 × 10^7^ to 5.1 × 10^9^ M^–1^ s^–1^. Given that both the Os­(II) donor complexes and the aromatic acceptors possess triplet excited states, the energy transfer is assigned to a Dexter EnT mechanism, involving short-range electron exchange and spin conservation. Recent experiments provide direct evidence of Dexter EnT in aqueous solution.^101^ Filipiak et al. demonstrated that ^3^CBBP* efficiently transfers its energy to 2-naphthalenesulfonate (NpSO_3_^–^) with a *k*_q_ of 2.8 × 10^9^ M^–1^ s^–1^, resulting in the formation of ^3^(NpSO_3_^–^)* as the dominant primary product, while radical anions such as CBBP^•–^ only appear on a slower time scale.^102^ This confirms that Dexter EnT can prevail over competing electron-transfer pathways, underscoring its mechanistic and environmental significance for pollutant activation.

As illustrated in Figure 8, Dexter-type triplet–triplet energy transfer can be described as a sequence of elementary steps that links molecular encounter dynamics to the observed quenching kinetics. ^3^Sens* and TC first diffuse together to form a solvent-shared encounter complex. Further collision and short-range orbital overlap may then generate a collision complex in which energy transfer can occur. This process is reversible. Dissociation of the collision complex can regenerate the encounter complex, which may either separate back to the original reactants or proceed to products.^103^ Therefore, the measured *k*_q_ reflects not only diffusion, but also the lifetime of the encounter/collision complex and the probability of energy transfer within that complex.^92^

**Figure 8:**
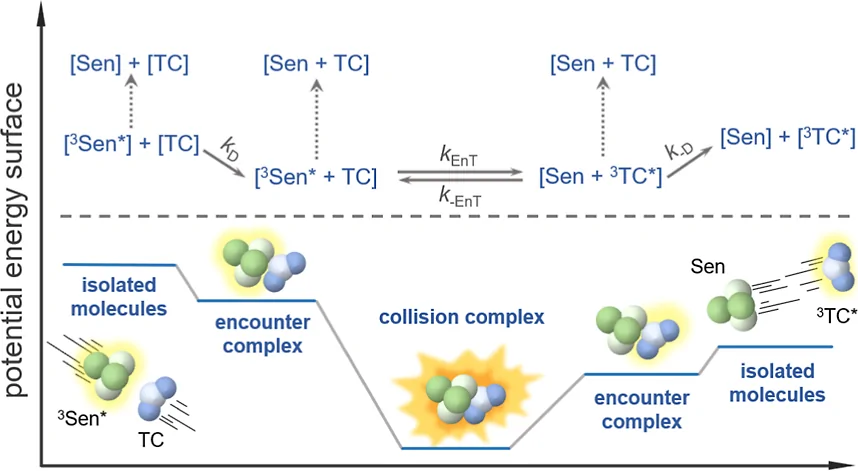
Representation of Dexter-type triplet–triplet energy transfer between ^3^Sens* and TC. The scheme illustrates diffusion, solvent-shared encounter-complex formation, short-range collision-complex formation, energy transfer through orbital overlap, and subsequent product separation or return to reactants. These steps provide a kinetic basis for understanding how diffusion, complex lifetime, and energy-transfer probability contribute to the observed quenching rate constant *k*_q_.

### 4.2 Redox-Based Quenching Pathways

#### 4.2.1 Photoinduced Electron Transfer

PET is a common quenching pathway for ^3^Sens* involving electron transfer triggered by the resonant interaction with electromagnetic radiation.^91^ Direct evidence for the PET comes from transient absorption spectra of short-lived intermediates.^104^ For example, in model systems with triplet pyridine derivatives (acting as a ^3^Sens*) and aniline, a broad absorption assigned to the aniline radical cation was observed, supporting PET where electron transfer occurs from ground-state aniline to ^3^Sens*.^105^

The relationship between *k*_q_ and the free energy change ( ${\Delta_{r}G}_{PET}^{ο}$ ) for PET can be evaluated using the Rehm and Weller equation^106,107^

11 $$k_{q}=\frac{k_{d}}{1+\frac{k_{d}}{K_{d}Z}\left\{\exp\left[\left(\sqrt{\frac{\Delta_{r}G_{PET}^{{}^{\circ}}}{2}+{\left(\frac{\lambda }{4}\right)}^{2}}+\frac{\Delta_{r}G_{PET}^{{}^{\circ}}}{2}\right)/RT\right]+\exp\left(\frac{\Delta_{r}G_{PET}^{{}^{\circ}}}{RT}\right)\right\}}$$

where *K*_d_ = *k*_d_/*k*_–d_ is the equilibrium constant for encounter complex formation, λ is the reorganization energy and *Z* is the universal collision frequency factor. ${\Delta_{r}G}_{PET}^{{}^{\circ}}$ can be calculated from electrochemical and photophysical parameters^108^

12 $${\Delta_{r}G}_{PET}^{{}^{\circ}}=e\times \left(E_{ox}^{{}^{\circ}}-E_{red}^{{}^{\circ}}\right)-E_{T}+w$$

Here, *e* is elementary charge, *E*_ox_^ο^ and *E*_red_^ο^ are the oxidation potential of the donor and the reduction potential of the acceptor, respectively, and *w* is the electrostatic interaction energy (approximately −0.02 eV in aqueous solution and often neglected). Many studies across various TCs show that for highly exergonic electron transfer reactions, *k*_q_ becomes diffusion-controlled and remains constant when ${\Delta_{r}G}_{PET}^{{}^{\circ}}$ is sufficiently negative.^14,109^ Figure 9 shows the dependence of *k*_q_ on ${\Delta_{r}G}_{PET}^{{}^{\circ}}$ , with a typical Rehm-Weller plot for three amino acids.^97^ In the endergonic region, *k*_q_ decreases rapidly with increasing ${\Delta_{r}G}_{PET}^{{}^{\circ}}$ , and log*k*_q_ exhibits linear relationships with ${\Delta_{r}G}_{PET}^{{}^{\circ}}$ depending on the amino acid donor type. This quantitative relationship provides a practical basis to predict PET feasibility for given ^3^Sen* and TC pairs, which is valuable when direct kinetic measurements for environmental pollutants are challenging.

**Figure 9:**
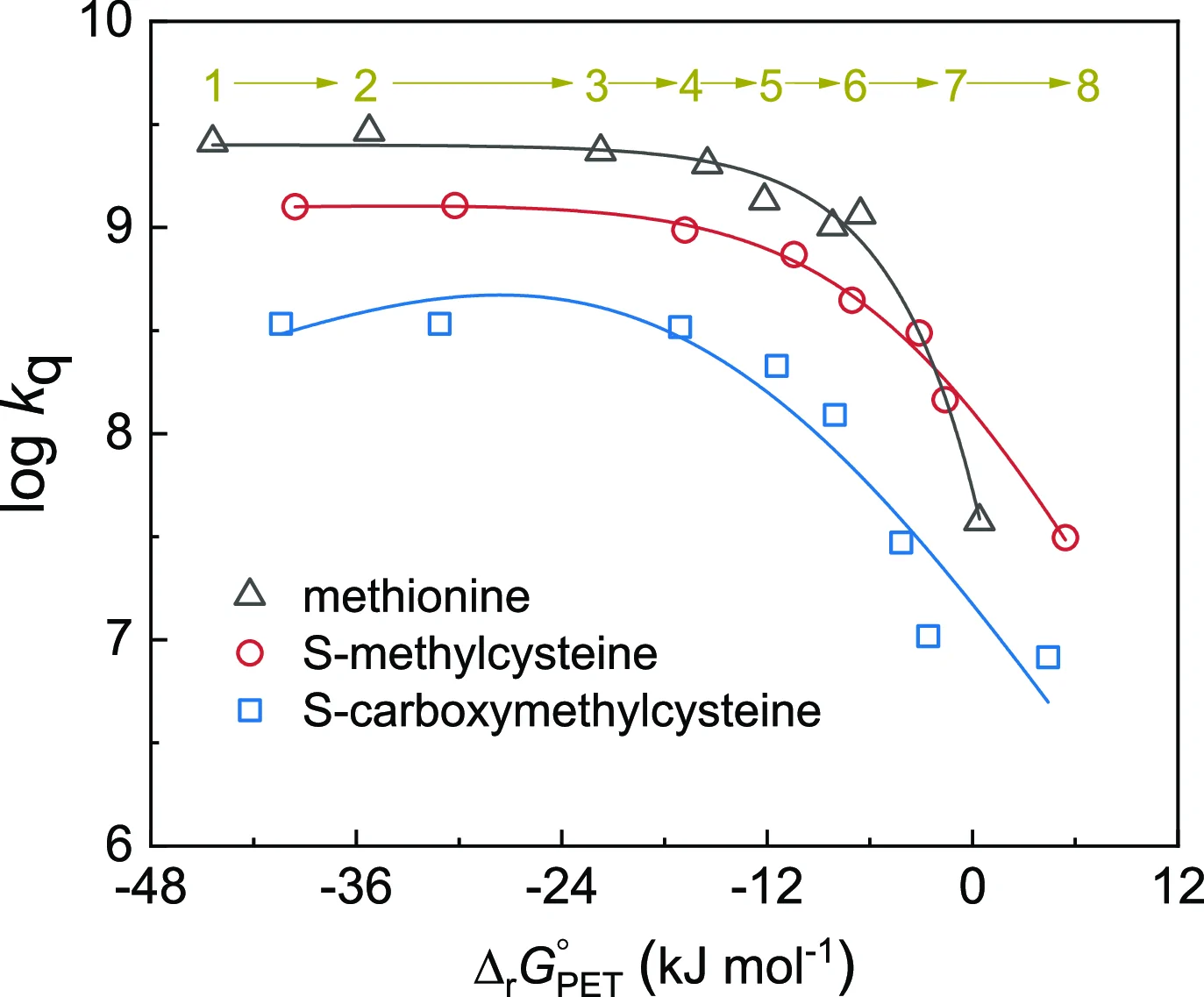
Dependence of *k*_q_ on Δ_r_ *G*_PET_^°^ for quenching of substituted benzophenone triplets by methionine, *S*-methylcysteine, and *S*-carboxymethylcysteine in water/acetonitrile solution (3:2 *v*/*v*) at pH 6.8. The numbers above the graph indicate the following ground-state benzophenones: (1) 3,3′-ditrifluoromethylbenzophenone, (2) 4-trifluoromethylbenzophenone, (3) 4-chlorobenzophenone, (4) benzophenone, (5) 4-methylbenzophenone, (6) 4,4′-dimethylbenzophenone, (7) 4-methoxybenzophenone, and (8) 4,4′-dimethoxybenzophenone. Reproduced from ref 109. Copyright 1993 American Chemical Society.

#### 4.2.2 Proton-Coupled Electron Transfer

PCET reactions involve simultaneous or sequential transfer of an electron and a proton between ^3^Sens* and TCs.^110,111^ In stepwise mechanisms, proton and electron transfers occur sequentially (proton first or electron first), while in concerted mechanisms, both transfer simultaneously without forming a stable intermediate. Distinguishing these pathways is often difficult. The kinetic isotope effect (KIE), measured by comparing quenching kinetics in H_2_O and D_2_O, is a common diagnostic to differentiate mechanisms. A representative example is the quenching of triplet kynurenic acid (^3^KNA*^–^) by *N*-acetyl-l-tryptophan (NTrpH).^112^ Transient absorption spectra revealed formation of a neutral radical (NTrp^•^) near 510 nm, indicating PCET. KIE measurements showed a pH-independent value of ∼1.3, helping clarify the mechanism. Since electron-transfer-initiated processes typically show low KIE (1.0–1.5)^113,114^ and concerted PCET typically yields KIE > 2.0,^115,116^ these results suggest quenching proceeds via initial electron transfer followed by proton transfer

13 KNA*−3+NTrpH→KNA·2−+NTrpH·+→KNAH·−+NTrp·

HAT is a subclass of PCET where the electron and proton transfer together from the same bond in one reactant.^117,118^ HAT mechanisms can also be confirmed via transient absorption spectra. For example, quenching of triplet thioxanthone (^3^TX*) by alcohols produces TXH^•^ and α-hydroxyalkyl radicals, indicating hydrogen abstraction.^119^ Reactivity correlates with α-C–H bond strength: alcohols with weaker α-C–H bonds (*e.g.*, benzyl alcohol) quench ^3^TX* more efficiently than methanol, which has a stronger bond. This correlation between *k*_q_ and α-C–H bond dissociation energies strongly supports a HAT pathway and highlights the role of bond energetics in hydrogen abstraction feasibility.

More generally, the bond dissociation energy (BDE) of the transferable X–H bond is a key descriptor for HAT-type quenching. A lower X–H BDE indicates that hydrogen atom abstraction is thermodynamically more favorable and usually leads to faster quenching, especially when the resulting radical is stabilized by resonance, heteroatoms, or adjacent π-conjugation. However, BDE alone does not fully determine HAT efficiency. Productive HAT also requires close molecular contact, favorable alignment between the X–H bond and the accepting excited-state orbital of ^3^Sens*, and sufficient accessibility of the transferable hydrogen atom. Therefore, X–H BDE is most useful when interpreted together with radical stability, steric effects, transient absorption evidence, kinetic isotope effects, and product analysis. In contrast, PET is more directly governed by electron-transfer free energy, whereas PCET additionally depends on p*K*_a_, proton availability, and hydrogen-bonding interactions. Thus, different redox-based pathways are controlled by different primary descriptors.

## 5 Implications and Future Directions

^3^Sens*-mediated photochemical transformations are widely recognized as important driving forces in environmental processes. While most studies investigate the roles of ^3^DOM*, this review consolidates the kinetic dimension of ^3^Sens*-mediated quenching, focusing on *k*_q_, which is critical for quantifying environmental pollutant fate.

Experimental techniques, namely LFP, PR, and TR-CIDNP, offer complementary capabilities. LFP excels in fast excited-state kinetics with high temporal resolution, PR is suited for radical-rich or less polar media, and TR-CIDNP resolves spectral overlap and enables indirect *k*_q_ determination. Each method bears limitations, including chromophore requirements, solvent interferences, and reaction specificity, which must be considered in data interpretation.

The *k*_q_ dataset (Table 2), though limited to dilute aqueous systems and predominantly organic TCs, provides a quantitative basis for comparing and predicting quenching kinetics. However, real environmental matrices, such as aerosols, cloud water, and saline or viscous media, exhibit substantial complexity and variability in pH, ionic strength, polarity, viscosity, and temperature. These parameters collectively modulate quenching through effects on molecular speciation, diffusion, and triplet lifetimes, profoundly influencing quenching behavior. This complexity remains insufficiently explored, representing a critical challenge for translating laboratory kinetics to natural systems and a priority for future research.

Molecular features such as electron-donating substituents, extended π-conjugation, and low steric hindrance generally enhance *k*_q_, particularly in electron-transfer-dominated quenching systems. These structure–reactivity relationships provide a basis for identifying TCs that are more likely to react rapidly with ^3^Sens* and for explaining differences in their quenching kinetics. Mechanistic evidence from TAS and KIE further distinguishes EnT, PET, PCET, and HAT pathways, thereby clarifying the kinetic origin of the observed reactivity differences. EnT is mainly controlled by molecular proximity, orbital overlap, and triplet-energy compatibility, whereas redox pathways depend strongly on electron-donating ability, molecular orbital energetics, free-energy changes, and bond strength. However, systematic kinetic datasets for matched substituent classes and positional isomers remain limited, and the individual contributions of electronic and steric effects are often difficult to separate. Further studies combining controlled molecular series with spectroscopic and theoretical analyses are therefore needed to establish more reliable structure–reactivity relationships and improve the prediction of TC quenching rates.

Future progress requires moving *k*_q_ measurements from idealized dilute solutions to matrices that better represent surface waters, cloud water, and aerosols, where pH, ionic strength, microviscosity, and temperature vary widely. Studies that report TC speciation together with Sens triplet lifetimes, and that vary these parameters in a controlled way, are needed to separate microenvironmental constraints from intrinsic EnT and redox reactivity. Methodologically, applying LFP, PR, and TR-CIDNP to matched Sens–TC pairs will strengthen cross-validation of *k*_q_ and improve confidence in mechanistic interpretation when transient signals overlap. Expanding datasets to mixed Sens systems (DOM fractions, particle-bound chromophores) and abundant inorganic quenchers (*e.g.*, halides) will be important for translating laboratory kinetics to real atmospheric and aquatic conditions.

## Nomenclature and Abbreviations

CO_3_^•–^: carbonate radicals
NpSO_3_^–^: 2-naphthalenesulfonate
O_2_^•–^: superoxide radicals
^•^H: hydrogen atom radicals
^•^OH: hydroxyl radicals
^1^O_2_: singlet oxygen
2-AN: 2-acetonaphthone
3-MAP: 3-methoxyacetophenone
AO: acridine orange
BP: benzophenone
BPTC: 3,3′,4,4′-benzophenone tetracarboxylic acid
CBBP: 4-carboxybenzophenone
(C)­DOM: (chromophoric) dissolved organic matter
DFX: difloxacin
DP: 2,2′-dipyridyl
FL: fluorenone
FMN: flavin mononucleotide
GMP: guanosine-5′-monophosphate
H_2_O_2_: hydrogen peroxide
KNA: kynurenic acid
MB: methylene blue
NATA: *N*-acetyl-l-tryptophanamide
NTrpH: *N*-acetyl-l-tryptophan
PAHs: polycyclic aromatic hydrocarbons
PhOH: phenol
R6G: rhodamine 6G
RB: rose bengal
RhB: rhodamine B
RZ: resazurin
SRHA: Suwannee River humic acid
Trp: tryptophan
TX: thioxanthone
Δ*A*: change of absorbance
***e***_aq_^–^: solvated electrons
EDGs: electron-donating groups
EnT: energy transfer
ESR: electron spin resonance
*E*_T_: triplet excitation energy
EWGs: electron withdrawing groups
FRET: Förster resonance energy transfer
HAT: hydrogen atom transfer
IC: internal conversion
HOMO: highest occupied molecular orbital
ISC: intersystem crossing
*J*: spectral overlap integral
*k*_0_: first-order decay rate constant in the absence of TC
*k*_D_: diffusion-controlled rate constant
*k*_obs_: pseudo-first-order decay rate constant
*k*_FRET_: the rate constant for FRET
KIE: kinetic isotope effect
*k*_Dexter_: the rate constant for Dexter EnT
*k*_q_: quenching rate constant
*k*_SV_: Stern–Volmer constant
LFP: laser flash photolysis
LINAC: linear accelerator
PR: pulse radiolysis
NMR: nuclear magnetic resonance
PCET: proton-coupled electron transfer
TR-CIDNP: time-resolved chemically induced dynamic nuclear polarization
PET: photoinduced electron transfer
LUMO: lowest unoccupied molecular orbital
RC: reference compound
RP: radical pair
RT: room temperature
Sens: photosensitizers
^3^Sens*: triplet-state photosensitizers
TCs: target compounds
TAS: transient absorption spectroscopy
TTA: triplet–triplet annihilation
DK: direct kinetics
